# Establishment of an orthotopic patient-derived xenograft mouse model using uveal melanoma hepatic metastasis

**DOI:** 10.1186/s12967-017-1247-z

**Published:** 2017-06-23

**Authors:** Ken Kageyama, Masahiro Ohara, Kengo Saito, Shinji Ozaki, Mizue Terai, Michael J. Mastrangelo, Paolo Fortina, Andrew E. Aplin, Takami Sato

**Affiliations:** 1 Department of Medical Oncology, Sidney Kimmel Cancer Center, Thomas Jefferson University, 1015 Walnut Street, Ste. 1024, Philadelphia, PA 19107 USA; 2 Department of Cancer Biology, Sidney Kimmel Cancer Center, Thomas Jefferson University, 1015 Walnut Street, Ste. 1024, Philadelphia, PA 19107 USA; 3 Department of Radiology, Osaka City University, 1-4-3 Asahimachi Abenoku, Osaka, Osaka 545-8585 Japan; 4 Department of Surgery, National Hospital Organization, Kure Medical Center/Chugoku Cancer Center, 3-1 Aoyamacho Kure, Hiroshima, 737-0023 Japan

**Keywords:** Patient-derived tumor xenograft, Uveal melanoma, Surgical orthotopic implantation, Liver, Mouse

## Abstract

**Background:**

Metastatic uveal melanoma is a highly fatal disease; most patients die from their hepatic metastasis within 1 year. A major drawback in the development of new treatments for metastatic uveal melanoma is the difficulty in obtaining appropriate cell lines and the lack of appropriate animal models. Patient-derived xenograft (PDX) tumor models, bearing ectopically implanted tumors at a subcutaneous site, have been developed. However, these ectopically implanted PDX models have obstacles to translational research, including a low engraftment rate, slow tumor growth, and biological changes after multiple passages due to the different microenvironment. To overcome these limitations, we developed a new method to directly transplant biopsy specimens to the liver of immunocompromised mice.

**Results:**

By using two metastatic uveal melanoma cell lines, we demonstrated that the liver provides a more suitable microenvironment for tumor growth compared to subcutaneous sites and that surgical orthotopic implantation (SOI) of tumor pieces allows the creation of a liver tumor in immunocompromised mice. Subsequently, 10 of 12 hepatic metastasis specimens from patients were successfully xenografted into the immunocompromised mice (83.3% success rate) using SOI, including 8 of 10 needle biopsy specimens (80%). Additionally, four cryopreserved PDX tumors were re-implanted to new mice and re-establishment of PDX tumors was confirmed in all four mice. The serially passaged xenograft tumors as well as the re-implanted tumors after cryopreservation were similar to the original patient tumors in histologic, genomic, and proteomic expression profiles. CT imaging was effective for detecting and monitoring PDX tumors in the liver of living mice. The expression of Ki67 in original patient tumors was a predictive factor for implanted tumor growth and the success of serial passages in PDX mice.

**Conclusions:**

Surgical orthotopic implantation of hepatic metastasis from uveal melanoma is highly successful in the establishment of orthotopic PDX models, enhancing their practical utility for research applications. By using CT scan, tumor growth can be monitored, which is beneficial to evaluate treatment effects in interventional studies.

**Electronic supplementary material:**

The online version of this article (doi:10.1186/s12967-017-1247-z) contains supplementary material, which is available to authorized users.

## Background

Uveal melanoma, which originates from the iris, ciliary body, or choroid, is highly fatal when it metastasizes. The mortality rate is over 90% within 2 years of initial diagnosis of metastasis, and median survival time ranges from 6 months to 1 year [1, 2]. Approximately 90% of metastatic uveal melanoma deaths are attributed to hepatic metastasis [2, 3]. Patients who first develop intra-hepatic metastasis have a shorter survival time than those who instead first develop extra-hepatic metastasis [4]. The major drawback in the development of new treatments for metastatic uveal melanoma is difficulty obtaining appropriate cell lines and animal models. In general, metastatic UM cell lines are very difficult to establish, and less than 20 metastatic uveal melanoma cell lines are currently available in the world. Furthermore, there are only a limited number of pre-clinical models using metastatic uveal melanoma to test the efficacy of a specific treatment. Currently available in vivo assays are either a subcutaneous injection of cell lines derived from uveal melanoma or retro-orbital injection of liver-selected murine cutaneous melanoma B16 cells [5, 6]. Given that uveal melanoma metastases typically colonize the liver and the genetics of uveal melanoma contrasts with that of cutaneous melanoma, there is a clear need to develop more biologically relevant in vivo models to test therapeutic strategies in advanced-stage uveal melanoma.

Patient-derived xenograft (PDX) tumor models, bearing implanted tumors from patients, were developed to meet the demands of precision medicine. A PDX mouse model holds promise because it offers a personalized treatment approach and may be useful to predict clinical prognosis, drug efficacy, and tumor characteristics [7]. Most PDX models, including previously reported uveal melanoma PDX models, are made by ectopic subcutaneous implantation due to the ease of this implantation technique [8, 9]. However, subcutaneous PDX models present fundamental limitations for translational research due to differences in the anatomic microenvironment from the hepatic site of tumor origin, low engraftment rate, and slow tumor growth [10–15]. Ideally, patient tumors should be implanted orthotopically into the same organ from which they were removed [16]. In the case of metastatic uveal melanoma, this is an orthotopic liver tumor xenograft model.

Orthotopic liver tumor xenograft mouse models were first described more than 2 decades ago [17]; however, these mouse models have not been well utilized due to technically demanding procedures for the establishment. The conventional implantation technique [18, 19] is complicated and requires the use of specialized equipment, including 6-0 to 8-0 fine suture under a microscope. Tumor and normal liver tissue must be sutured carefully so that the suture does not injure the fragile liver tissue and lead to hematoma [20]. To circumvent these technical difficulties, we developed a novel surgical orthotopic implantation (SOI) technique in which a pocket is made in the liver parenchyma to houses the tumor entirely within the parenchyma (liver pocket method), followed by the closure of incision site with absorbable hemostatic materials, instead of the suture. Our method provides an optimal microenvironment for tumor development in the liver and it is especially suitable for hepatic metastasis from primary uveal melanoma.

As described above, the main purpose of this study is to circumvent the limitations of conventional PDX models that use subcutaneous implantation by developing an appropriate orthotopic hepatic PDX model of metastatic uveal melanoma in the NOD.Cg-Prkdc^scid^ Il2rg^tm1Wjl^/SzJ (NSG) mouse. To develop orthotopic hepatic transplant models, we first compared the differences in tumor cell growth in a liver site versus a subcutaneous site using two metastatic uveal melanoma cell lines, to ascertain which site provides a more suitable tumor microenvironment for the tumor growth. Then, the feasibility of SOI of tumor tissues (liver pocket method) was investigated to develop a practical liver tumor xenograft mouse model for metastatic uveal melanoma. Finally, by using this method, we attempted to establish orthotopic PDX mouse models from hepatic metastasis samples from 12 different uveal melanoma patients. We evaluated concordance for tumor characteristics between pre-implanted and post-implanted tumors. To our knowledge, our study is the first demonstration of an orthotopic hepatic PDX transplant model for metastatic uveal melanoma.

## Results

### Liver is a suitable microenvironment to support growth of metastatic uveal melanoma cell lines

**Fig. 1:**
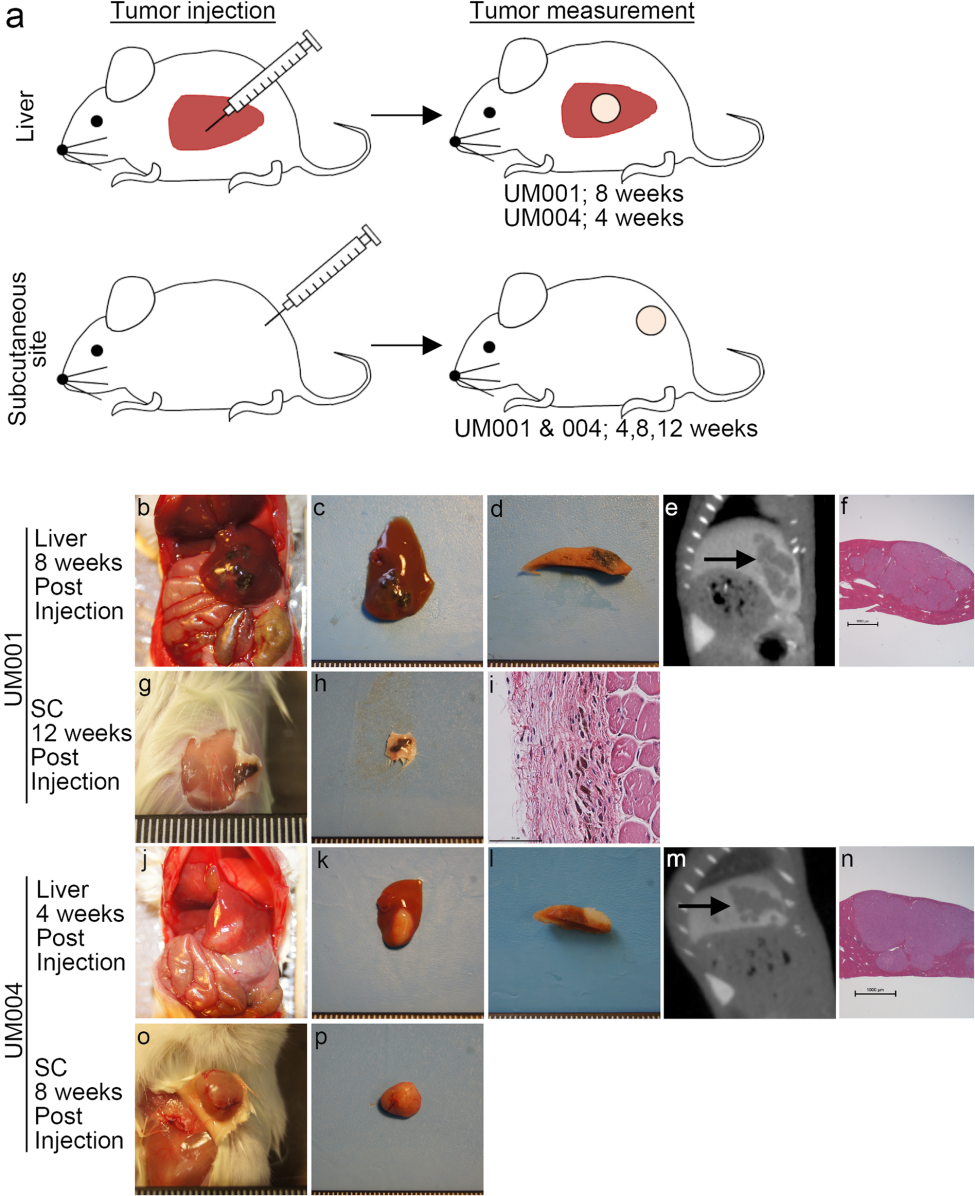
**a** Schematic overview of liver and subcutaneous tumor implantation by needle injection using established cell lines. For mice injected with tumor suspensions into the liver, tumor volume was measured at sacrifice. Liver UM001 tumor-bearing mice were sacrificed at 8 weeks and UM004 tumor-bearing mice were sacrificed at 4 weeks post-injection. For mice injected with tumor suspensions at the subcutaneous site, tumor volume was measured three times (4, 8, and 12 weeks) before the mice were sacrificed at 12 weeks post-injection. **b**–**p** Macroscopic, histopathological, and radiological features of liver and subcutaneous tumors generated with established cell lines. **b**, **j** Laparotomy image. **c**, **h**, **k**, **p** Macroscopic findings for resected tumors. **d**, **l** Cut surface of the tumor. **e**, **m** Sagittal imaging using CT, *black arrows* indicate tumors. **f**, **n** H&E staining, ×10, *Scale bar* 1 mm. **g**, **o** Macroscopic findings for subcutaneously injected sites. **i** H&E staining, ×400, *Scale bar* 50 µm. *SC* subcutaneous site

To compare growth characteristics of human uveal melanoma cells at hepatic and subcutaneous xenograft sites in the mouse, TJU-UM001 and TJU-UM004 single cell suspensions were injected at 1 × 10^6^ cells into the liver and the subcutaneous site, respectively (Fig. 1). At 8 weeks after injection, UM001 tumors were confirmed to be engrafted in the liver in three mice (Table 1). In contrast, the subcutaneous site did not engraft the UM001 tumors, although mice were observed for up to 12 weeks after injection. UM004 tumors grew at both the liver and subcutaneous sites; however, UM004 tumors were significantly larger in the liver than the subcutaneous site at 4 weeks after injection. To re-examine whether UM001 cells can grow at the subcutaneous site, three different cell titers (2, 5, and 10 × 10^6^ cells) of UM001 cells were injected, but UM001 tumors did not form by 12 weeks post injection. Only the cicatrix of a tumor was found at 12 weeks when a higher number of tumor cells was injected into the subcutaneous site. These findings indicate that compared to the subcutaneous site, the liver offers a more suitable microenvironment to grow the metastatic uveal melanoma cell lines.

**Table 1:** Tumor engraftment at liver and subcutaneous implantation sites using human uveal melanoma cell lines

Method	Cell lines	Cell number (×10^6^)	Tumor chunk (mm^3^)	Injection site	Mouse number	Tumor engraftment rate (median tumor volume, range) (mm^3^)			Metastasis		Ki67 % positive cells (median, range)
						4 weeks	8 weeks	12 weeks	Intra-hepatic	Extra-hepatic	
Needle injection	UM001	1	NA	Liver	3	NA	3/3 (123.8, 73.7–126.0)	NA	0/3^a^	0/3	44.9, 42.6–48.2
		1	NA	SC	3	0/3	0/3	0/3	0/3	0/3	NA
		2	NA	SC	3	0/3	0/3	0/3	0/3	0/3	NA
		5	NA	SC	3	0/3	0/3	0/3^b^	0/3	0/3	NA
		10	NA	SC	3	0/3	0/3	0/3^b^	0/3	0/3	NA
	UM004	1	NA	Liver	3	3/3 (225.2, 102.8–259.0)	NA	NA	0/3^a^	0/3	85.8, 82.5–90.4
		1	NA	SC	3	3/3 (0.49, 0.08–0.6)	3/3 (852.5, 465.4–1418.4)	NA^c^	0/3	0/3	77.4, 64.0–80.1
Surgical implantation	UM001	NA	1	Liver	5	NA^d^	NA	NA	0/5	0/5	18.2, 10.3–28.9
		NA	1	Liver	5	NA	5/5 (153.7, 150.7–251.3)	NA	0/5	0/5	42.6, 30.3–56.8
		NA	1	SC	5	NA	5/5 (1.12, 0.32–2.25)	NA	0/5	0/5	14.1, 7.2–18.5
	UM004	NA	1	Liver	5	NA^d^	NA	NA	0/5	0/5	46.1, 28.1–64.0
		NA	1	Liver	5	5/5 (309.3, 253.9–518.3)	NA	NA	0/5	0/5	88.5, 80.9–96.4
		NA	1	SC	5	5/5 (21.9, 21.43–28.11)	NA	NA	0/5	0/5	44.1, 25.4–57.9

*SC* subcutaneous site, *NA* not applicable

^a^Daughter nodules appeared around the main tumor. Metastatic tumors did not emerge in the other lobe

^b^Only pigment was visually detected at sacrifice; there were no measurable tumors

^c^All mice were sacrificed earlier than 12 weeks post-implantation, because tumor diameter reached 15 mm or more

^d^These mice were sacrificed at 1 day post implantation

### Surgical orthotopic implantation (SOI) using the liver pocket method generates a liver tumor xenograft model

**Fig. 2:**
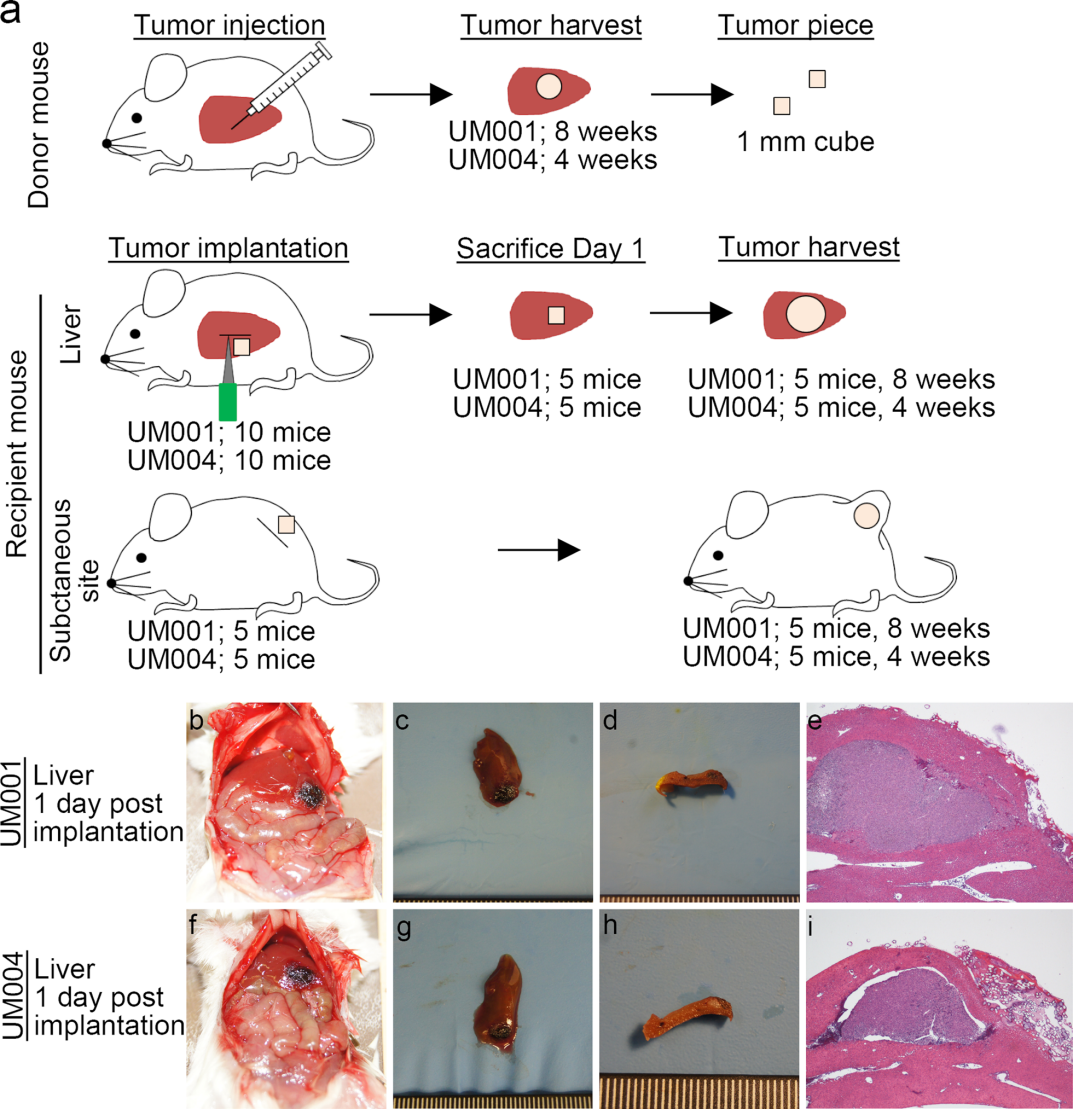
**a** Schematic overview of the surgical implantation of tumor pieces into the liver and a subcutaneous site. Tumor mass was generated in donor mice following injection of UM001 and UM004 cell lines to the liver, as described in Fig. 1a. Tumor mass was harvested from the donor mouse and cut into 1 mm cubes. These small tumor pieces were transplanted into the liver or a subcutaneous site of recipient mice. UM001- and UM004-derived tumor pieces were separately implanted into the liver in 10 mice for each cell line. Five mice were sacrificed at 1 day after implantation. The remaining five mice were sacrificed at 8 weeks after implantation for UM001, or 4 weeks after implantation for UM004, respectively. Furthermore, UM001- and UM004-derived tumor pieces were also separately implanted into the subcutaneous site in five mice for each cell line. The five mice were sacrificed at the same time points as the liver tumor-bearing mice. **b**–**i** Histopathological features of implanted tumors in the liver at 1 day post implantation using established cell lines. **b**, **f** Laparotomy image. **c**, **g** Macroscopic findings for resected tumor. **d**, **h** Cut surface of the tumor. **e**, **i** H&E staining, ×40, *Scale bar* 500 µm

To develop a practical liver tumor xenograft model, a novel procedure for SOI of the tumor piece (liver pocket method) was developed. Donor tumors, which were generated in mouse host livers by injecting uveal melanoma cell lines, were surgically transplanted into the livers of recipient mice (Fig. 2). This new SOI with liver pocket method was successfully performed on all 20 recipient mice, with ten receiving UM001 tumor pieces and ten receiving UM004 tumor pieces (Additional file 1: Table S1). Median operation time was 22 min, and median blood loss during the operation was 0.13 g. Adverse events, such as hematoma, bile leakage, and tumor deviation, did not occur in any of the 20 mice. Half of the 20 mice were sacrificed at day 1 post-implantation (5 UM001 mice and 5 UM004 mice). An autopsy confirmed that tumors remained localized in the parenchyma of the liver without any tumor deviation in these ten mice (Fig. 2). The remaining ten mice were sacrificed later (five UM001 mice at 8 weeks post-implantation and five UM004 mice at 4 weeks post-implantation). For comparison with the liver implanted tumors, tumor pieces generated from the uveal melanoma cell lines were also implanted at the same time into the subcutaneous site in ten additional mice (five mice for UM001 and five mice for UM004).

As shown in Table 1 and Additional file 2: Figure S1, all recipient mice in which tumor pieces were directly transplanted into the liver using the liver pocket method developed tumors in their liver. By comparison, the mice in which tumor pieces were implanted subcutaneously exhibited much smaller tumors at the same respective time points. Neither of the mouse groups developed tumors anywhere except at the implanted sites. The ratio of Ki67-positive cells was higher in the liver tumors than in the subcutaneous tumors for both cell lines tested (UM001 recipient liver vs. recipient subcutaneous site, p < 0.001; UM004 recipient liver vs. recipient subcutaneous site, p < 0.001) (Table 1).

### Concordance for tumor characteristics between donor and recipient tumors in the liver

The expression of three different melanoma markers (HMB45, MelanA, and S100) and a cell proliferation marker (Ki67) was similar between donor and recipient tumors generated from the UM001 and UM004 cell lines (Additional file 3: Figure S2a). The ratio of Ki67-positive cells in the liver was transiently decreased at 1 day post-implantation; however, the ratio of Ki67-positive cells was similar between donor and established recipient liver tumors (UM001 donor liver vs. recipient liver, p = 0.785; UM004 donor liver vs. recipient liver, p = 0.786) (Table 1). By reverse phase protein array (RPPA) analysis, both donor and recipient liver tumors showed highly matching tumor characteristics with respect to protein expression, yielding Pearson correlation analysis results of r^2^ = 0.9559, p < 0.001 for UM001 tumors and r^2^ = 0.8741, p < 0.001 for UM004 tumors, respectively (Additional file 3: Figure S2b, c).

### Establishment of an orthotopic patient-derived liver metastatic uveal melanoma xenograft model

**Fig. 3:**
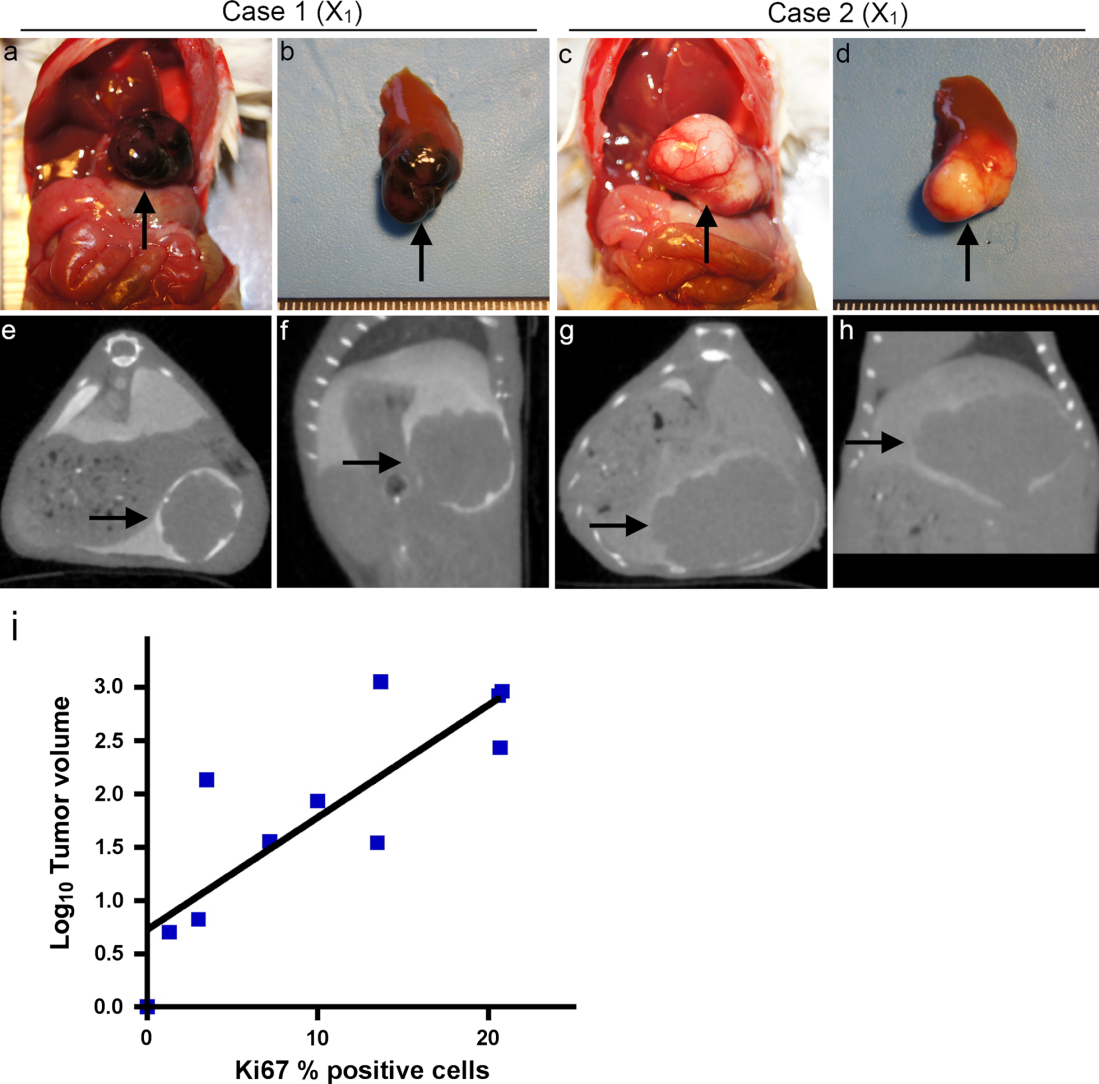
**a**–**h** Two representative examples of the histopathological and radiological features of the uveal melanoma PDX models. **a**, **c** Laparotomy. **b**, **d** Macroscopic findings for resected liver, showing left lobe with tumor. **e**, **g** Axial imaging on CT. **f**, **h** Sagittal imaging on CT. *Black arrows* indicate patient-derived xenograft tumors. **i** Correlation between the ratio of Ki67-positive cells in original patient tumors and median tumor volume in first-generation mice bearing the corresponding patient-derived tumors. y = 0.1054x + 0.725, r = 0.836, r^2^ = 0.699, p < 0.01. Linear scale is converted to logarithmic scale for tumor volume

After establishing the SOI method in NSG mice, we investigated whether an orthotopic PDX mouse model can be established using hepatic metastasis specimens from actual uveal melanoma patients. Patient-derived tumor specimens were obtained by core biopsy (n = 10) or surgery (n = 2) from 12 uveal melanoma patients with hepatic metastasis. These patient-derived tumor specimens were implanted to the liver of NSG mice, and the NSG mice were observed up to six months. All mice were sacrificed at 6 months post-tumor implantation after contrast-enhanced CT imaging evaluation. As shown in Table 2; Fig. 3a–h and Additional file 4: Figure S3, 10 PDXs were established successfully from the 12 patients (engraftment rate 83.3%) within 6 months. Overall, tumors were confirmed in 18 of 27 implanted mice (66.7%). The presence of tumors was confirmed by contrast enhanced CT imaging in all cases. It is of note that needle biopsy specimens successfully engrafted in 8 of 10 patients (80%). Since hepatic metastasis tends to develop in both lobes of the liver, surgical removal of the tumor is not a feasible option for the majority of patients. In this regard, the successful PDX tumor establishment from core biopsy specimens is highly encouraging.

**Table 2:** Results for generation of liver orthotopic patient-derived xenograft mouse models and their tumor characteristics

Case	Patient tumor (X_0_)			Xenograft (X_1_)			Xenograft (X_2_)	Xenograft (X_3_)	Pigmentation		Ki67% positive cells (%)		Monosomy 3^a^		c-myc copy number^a^		GNAQ		GNA11		BAP1		SF3B1		EIF1AX	
	Sex	Age	Sample	Engraftment	Tumor take rate (mouse)	Volume (mm^3^) (median, range)	Engraftment	Engraftment	X_0_	X_1_	X_0_	X_1_	X_0_	X_1_	X_0_	X_1_	X_0_	X_1_	X_0_	X_1_	X_0_	X_1_	X_0_	X_1_	X_0_	X_1_
1	F	59	Surgery	Success	3/3	272.2, 240.3–344.6	Success	Success	Moderate	Moderate	20.7	16.1	+	+	Large	Large	–	–	–	–	+	+	–	–	–	–
2	F	41	Biopsy	Success	2/2	931.8, 831.7–1031.9	Success	Success	None	None	20.8	20.1	+	+	Large	Large	+	+	–	–	–	–	–	–	–	–
3	M	67	Biopsy	Failure	0/3	NA	NA	NA	None	NA	IS	NA	+	NA	Large	NA	IS	NA	IS	NA	IS	NA	IS	NA	IS	NA
4	M	70	Biopsy	Success	2/2	86.73, 60.0–113.4	Success	Success	Moderate	Moderate	10.0	14.3	+	+	Large	Large	+	+	–	–	–	–	–	–	–	–
5	F	59	Biopsy	Success	2/2	835.3, 572.9–1097.7	Success	Success	None	None	20.6	24.6	+	+	Large	Large	–	–	+	+	+	+	–	–	–	–
6	F	62	Biopsy	Failure	0/2	NA	NA	NA	Heavy	NA	0	NA	–	NA	Small	NA	–	NA	–	NA	–	NA	+	NA	–	NA
7	M	66	Biopsy	Success	3/3	35.3, 28.2–199.1	Success	Success	None	Minimal	13.5	16.1	+	+	Small	Small	–	–	+	+	+	+	–	–	–	–
8	M	73	Biopsy	Success	2/2	137.8, 123.0–152.6	Failure	NA	None	Minimal	3.5	4.5	IS	+	IS	Large	IS	IS	IS	IS	IS	IS	IS	IS	IS	IS
9	M	71	Surgery	Success	1/4	36.1	Failure	NA	Moderate	Moderate	7.2	3.9	+	+	Large	Large	–	–	+	UR	+	+	–	–	–	–
10	F	49	Biopsy	Success	1/1	6.61	Failure	NA	Minimal	Minimal	3.0	6.9	–	–	Small	Small	+	+	–	–	–	–	+	+	–	–
11	F	52	Biopsy	Success	2/3	5.08, 4.85–5.32	Failure	NA	Moderate	Moderate	1.3	0	IS	+	IS	Small	–	–	–	–	–	–	–	–	–	+
12	M	48	Biopsy	Success	1/1	1123.7	Success	Success	Moderate	Moderate	13.7	18.3	+	+	Large	Large	+	+	–	–	+	+	–	–	–	–

*X*_*0*_ patient’s tumor, *X*_*1*_ first-generation mice, *X*_*2*_ second-generation mice, *X*_*3*_ third-generation mice, *F* female, *M* male, *NA* not applicable, *IS* insufficient samples, *UR* unreadable due to low coverage of this lesion, *none* no pigmentation, *minimal* one-third or less pigmented, *moderate* one-third to two-thirds pigmented, *Heavy* two-thirds to complete pigmented, *large* 4 copies, *small* 3 copies or partial 4 copies

^a^Monosomy 3 and c-myc gain were evaluated by copy number variation analysis

To maintain PDX tumors in NSG mice, tumors obtained from the first generation of mice (X1) were serially transplanted to the next cohorts of mice (X2 and X3). All 10 xenograft tumors were successfully transplanted into second-generation mice (X2). We were able to serially passage 6 of 10 PDX tumors and engraft them into third-generation mice (X3). By examining the expression profiles of the original patient tumors, we found that high expression of Ki67 was a predictive factor for their ability to undergo serial passaging and successful engraftment into the third-generation mice (p < 0.05) (Table 3). The ratio of Ki67-positive cells in original patient tumors correlated significantly with median tumor volume in first-generation mice (Pearson correlation analysis of r^2^ = 0.699, p < 0.01; Fig. 3i). Collectively, these results indicate that high expression of Ki67 in parental tumors confers enhanced tumor growth and successful serial passages in PDX mice.

**Table 3:** Predictive factors for successful engraftment of PDX tumor

	Engraftment of 1st-generation mice (X_1_)		p value	Achievement of serial passages to 3rd-generation mice (X_3_)		p value		Engraftment of 1st-generation mice (X_1_)		p value	Achievement of serial passages to 3rd-generation mice (X_3_)		p value
	Success	Failure		Success	Failure			Success	Failure		Success	Failure	
Patient gender	n = 12			n = 12			c-myc gain	n = 10			n = 10		
Male	5	1	1.00	3	3	1.00	High gain	6	1	1.00	5	2	0.500
Female	5	1		3	3		Low gain	2	1		1	2	
Age	n = 12			n = 12			GNAQ	n = 10			n = 10		
≥60	4	2	0.454	2	4	0.567	Positive	4	0	1.00	3	1	0.571
<60	6	0		4	2		Negative	5	1		3	3	
Tumor sample	n = 12			n = 12			GNA11	n = 10			n = 10		
Surgery	2	0	1.00	1	1	1.00	Positive	3	0	1.00	2	1	1.00
Biopsy	8	2		5	5		Negative	6	1		4	3	
Pigmentation	n = 12			n = 12			BAP1	n = 10			n = 10		
None	4	1	1.00	3	2	1.00	Positive	5	0	1.00	4	1	0.528
Pigments	6	1		3	4		Negative	4	1		2	3	
Ki67 (%)	n = 11			n = 11			SF3B1	n = 10			n = 10		
≥10	6	0	0.455	6	0	0.002	Positive	1	1	0.200	0	2	0.133
<10	4	1		0	5		Negative	8	0		6	2	
Monosomy 3	n = 10			n = 10			EIF1AX	n = 10			n = 10		
Positive	7	1	0.378	6	2	0.133	Positive	0	0	1.00	0	0	1.00
Negative	1	1		0	2		Negative	9	1		6	4	

*Large* 4 copies, *Small* 3 copies or partial 4 copies

### Reimplantation of cryopreserved tumors

After successful engraftment of patient-derived xenograft tumors in first-generation mice (X1), three 1-mm cubes of chunks from individual PDX tumors obtained from cases 2, 5, 7, and 12 were cryopreserved in liquid nitrogen using three different cryopreservation media (F, D, R medium respectively). After cryopreservation, these cryopreserved PDX tumor specimens were re-implanted. All cryopreserved PDX tumor chunks except one in F medium from case 7 were successfully engrafted into the livers of recipient mice, as were freshly transplanted tumor pieces without cryopreservation (Table 4). Tumor volumes of the cryopreserved tumors were smaller than those of the directly transplanted tumors when they were sacrificed at 6 months after tumor transplantation. Among the three freezing methods tested, Cryomedium D (DMEM containing medium) resulted in the highest growth rates following thawing and re-implantation of the frozen tumor samples. Despite the slower growth of the cryopreserved tumor specimens, the ratio of Ki67-positive cells in the cryopreserved tumors was similar to that of the directly transplanted tumors. From these studies, we conclude that while cryopreserved tumors can be successfully engrafted in mice, the growth of cryopreserved tumor samples is reduced compared to the directly transplanted, non-frozen tumor samples. Considering comparative Ki67 activity in established PDX tumor specimens in X2 mice, this is most likely due to loss of a fraction of proliferative tumor cells in the cryopreserved tumor chunks.

**Table 4:** Results for re-implantation of cryopreserved tumors

Case	Pre-implanted tumor			Cryo-medium	Cryopreserved days	Engraftment	Post-implanted tumor				
	Generation	Pigment	Ki67% positive cells (%)				Generation	Tumor take rate (mouse)	Volume (mm^3^)	Pigment	Ki67% positive cells (%)
2	X_1_	None	20.1	Fresh	None	Success	X_2_	1/1	958.9	None	17.0
				F	30	Success		1/1	189.2	None	15.4
				D		Success		1/1	364.1	None	15.0
				R		Success		1/1	103.5	None	14.9
5	X_1_	None	24.6	Fresh	None	Success	X_2_	1/1	1258.0	None	22.8
				F	15	Success		1/1	128.3	None	14.2
				D		Success		1/1	635.1	None	19.8
				R		Success		1/1	254.3	None	17.3
7	X_1_	Minimal	16.1	Fresh		Success	X_2_	1/1	365.1	Minimal	15.9
				F	15	Failure		0/1	–	–	–
				D		Success		1/1	5.5	Minimal	15.4
				R		Success		1/1	0.32	Minimal	15.7
12	X_1_	Moderate	18.3	Fresh	None	Success	X_2_	1/1	998.5	Moderate	20.4
				F	15	Success		1/1	92.34	Moderate	15.6
				D		Success		1/1	686.7	Moderate	16.9
				R		Success		1/1	502.1	Moderate	15.2

*X*_*0*_ patient’s tumor; *X*_*1*_ first-generation mice, *X*_*2*_ second-generation mice, *fresh* direct implantation without cryopreservation, *F* F medium (90% fetal bovine serum/10% DMSO), *D* D medium (70% DMEM/20% fetal bovine serum/10% DMSO), *R* R medium (70% RPMI/20% fetal bovine serum/10% DMSO)

### Xenograft tumors histologically resemble the original hepatic metastasis obtained from patients

**Fig. 4:**
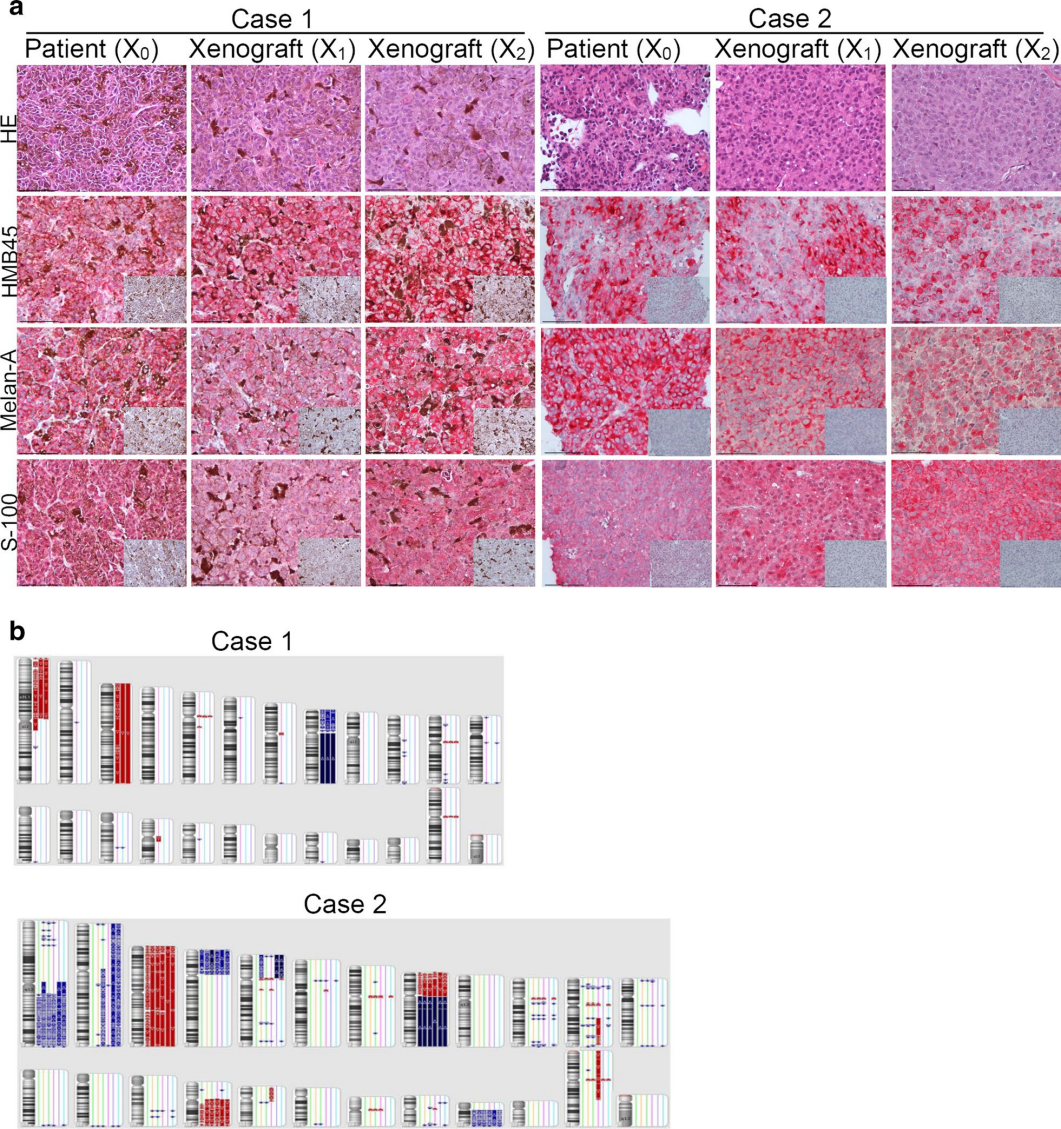
**a** Two representative examples of histopathological features of patient tumors and the corresponding xenograft tumors. H&E-stained sections and immunostained sections with HMB-45, Melan A, and S-100 antibodies, ×400. *Scale bar* 50 µm. Patient tumors are depicted in the *columns 1* and *4*. The corresponding xenograft tumors are depicted in *columns* *2* and *3*, and *5* and *6*, respectively. *Control panels* show no positive signal with an isotype control antibody (*inset* images located at *bottom right* in each immunostaining* panel*). *X*_*0*_ Patient original tumors, *X*_1_ PDX tumors in the first-generation mice, *X*_*2*_ PDX tumors in the second-generation mice. **b** Two representative examples of DNA copy number variation with karyogram. Individual chromosomes are shown in the karyograms, with *bars* on the *right side* of the karyograms indicating the chromosomal locations of copy number losses and gains, respectively. Chromosomes 1 to 12 are lined up on the *top* and chromosome 13 to 22, *X* and* Y* are on the *bottom*. From *left* to *right*, patient tumor (X_0_) is at the* left* with its adjacent karyogram, the corresponding first-generation xenograft tumor (X_1_) is in the* second column*, the corresponding second-generation xenograft (X_2_) is in the* third column*, and three different xenograft tumors generated from frozen tumor specimens using F, D and R cryopreservation medium are in the *fourth* to *sixth columns*. A copy number of 2 (normal) is indicated by *blank space* (*no color*); copy number greater than 2 (chromosomal gain or amplification) is indicated in *blue*; and copy number less than 2 (chromosomal loss or deletion) is indicated in *red*

Hematoxylin and eosin (H&E) staining revealed a strong concordance between the histopathological features of the patient tumors and their corresponding xenograft tumors in mice (Fig. 4a and Additional file 5: Figure S4). Xenograft tumors retained a similar degree of pigmentation as observed in the original patient tumors (Table 2). Serially passaged xenograft tumors also retained the same morphology and pigmentation as their original patient tumors. The three melanoma markers (HMB-45, Melan-A, and S100) were expressed at similar relative levels in patient tumors and their corresponding xenograft tumors. Furthermore, the ratio of Ki67-positive cells in the original tumors was also akin to that in the corresponding xenograft tumors.

### Xenograft tumors retain mutations present in their parental patient tumors

We identified five representative mutations in uveal melanoma. In 10 out of 12 samples available for mutation analysis, the xenograft tumors contained the exact same mutations as their corresponding original patient tumors. An exception was observed for case 11 at the EIF1AX locus (Table 2). The mutation rate for five representative mutations was 40, 30, 50, 20, and 10% in GNAQ, GNA11, BAP1, SF3B1, and EIF1AX, respectively. Mutations in GNAQ and GNA11 were mutually exclusive, as were mutations in BAP1, SF3B1, and EIF1AX with each other. For PDX cases 2, 5 and 12, a total of nine tumor samples re-implanted after cryopreservation also retained the same mutational profiles as their corresponding original patient tumors.

### Xenograft tumors preserve the DNA copy number alterations in their original patient tumors

**Fig. 5:**
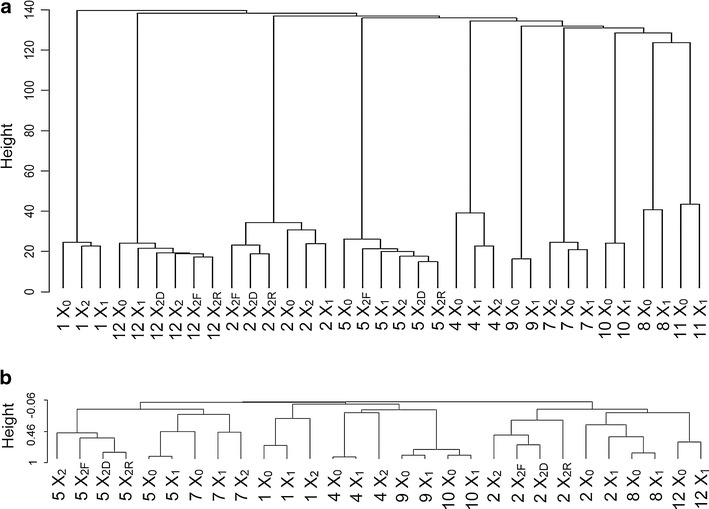
**a** Unsupervised hierarchical clustering of paired patient tumors and xenograft tumors based on DNA copy number analysis. *X*_*0*_ Patient original tumors, *X*_*1*_ PDX tumors in the first-generation mice, *X*_*2*_ PDX tumors in the second-generation mice, *F* F medium (90% fetal bovine serum/10% DMSO), *D* D medium (70% DMEM/20% fetal bovine serum/10% DMSO); *R* R medium (70% RPMI/20% fetal bovine serum/10% DMSO). **b** Unsupervised hierarchical clustering of paired patient tumors and xenograft tumors based on reverse phase protein array (RPPA) analysis

The patterns of copy number variations (CNVs) in the original patient tumors are mostly maintained in the corresponding first-, second-, and third-generation xenograft tumors, as well as in tumors generated by re-implantation of cryopreserved samples (Fig. 4b; Additional file 6: Figure S5). CNVs in tumors displayed representative characteristics of uveal melanoma cells, including monosomy 3 accompanied by chromosome 1p loss, 8q gain, and 8p loss. In particular, the 8q gain was observed in all 12 cases, as was c-myc amplification in 8q. For cases 8 and 11, CNVs in patient tumors did not match the corresponding original tumors. These patient tumors showed a normal pattern of CNVs because of the very small volumes of tumor in the biopsy specimen, whereas the xenograft tumors displayed a typical pattern of uveal melanoma CNVs. Unsupervised hierarchical clustering using patterns of CNV revealed that patient tumors clustered with the corresponding xenograft tumors and the corresponding cryopreserved tumors in 10 successful engraftment cases (Fig. 5a).

### Stability of xenograft tumors on their proteome as determined by reverse phase protein array (RPPA)

We performed RPPA on nine paired original patient tumors and the corresponding xenograft tumors for ten successful engraftment cases (excluding case 11 due to unavailability of tumor samples). The related samples clustered together regarding their protein expression profiling, thus demonstrating stability at the level of the proteome between the original patient tumors and their subsequently derived PDX tumors (Fig. 5b).

## Discussion

To generate PDX mouse models, the ectopic subcutaneous implantation method has been used due to quick implantation of tumors and ease of monitoring [8]. However, orthotopic engraftment of the tumor is more rational for a PDX model because tumor cells reside in the similar microenvironment as the original tumor [21, 22]. For example, orthotopic soft-tissue sarcoma xenografts retained the same morphology as primary tumors, whereas ectopically implanted xenograft tumors altered their morphology due to the influence of the tumor microenvironment [23]. Orthotopic breast tumor xenografts were considered to be more accurate models for tumor microenvironment and clinical cancer progression compared with the subcutaneous xenografts [24]. Similarly, lung and colon cancer xenograft mouse models respond differently to chemotherapy, depending on the sites of transplantation [25, 26]. Collectively, these studies indicate that patient-derived orthotopic xenograft (PDOX) tumor models mirror the corresponding patient tumors more accurately than models using ectopic implantation sites [16].

Our liver pocket method makes surgical orthotopic implantation (SOI) in the liver simpler than the conventional techniques, allowing us to finish implantation of patient tumor tissues into the liver in less than 5 min, most of which time is required for closing the abdomen with a double-layer suture. Absorbable hemostatic material plays a major role in sealing the incision and preventing bleeding. Average bleeding volume was approximately less than 10% of circulating blood volume in mice [27]. Because the hemostatic materials prevented surgical bleeding, we were able to operate with greater confidence and achieve excellent survival outcomes for our PDX mouse models without treatment-related death.

Although PDX models have proven to be useful for cancer research, there are still some obstacles to their use in translational research [7, 28, 29]. First, engraftment failure occurs at a high rate for some tumors, including subcutaneous implantation of uveal melanoma [9, 13, 14]. It is essential to improve tumor engraftment so that a larger number of PDX models can be successfully generated, enabling them to be effectively utilized for tumor data profiling and mouse drug efficacy trials, and avoiding selection bias of PDX models due to poor engraftment rate. In our study, we found that the orthotopic hepatic implantation technique enhanced the rate of tumor engraftment of metastatic uveal melanoma, compared to subcutaneous implantation with uveal melanoma metastases [9]. Additionally, most PDX studies have required surgically harvested tumor materials, not needle biopsy specimens [7] to obtain sufficient amounts of tumor specimens from patients. Engraftment failure is commonly encountered in core biopsy samples containing fewer tumor cells. In this regard, the high success rate of engraftment in our method is encouraging and suggests that core biopsy specimens can be used effectively to generate PDX models by orthotopic implantation.

One of the disadvantages of PDX models for drug screening is the discrepancy between engraftment time in mice and imminent treatment schedules for patients, which limits timely personalized pre-clinical drug trials using PDX models [7, 28]. Our study harvested the tumors in less than 6 months post-implantation, which is shorter than the time required for tumor establishment in subcutaneous implantation models; however, additional time is still needed to generate second- or third-generation PDX mouse models for personalized drug trials. We plan to investigate whether we can develop PDX tumors from primary uveal melanoma. If this approach is successful, we might be able to develop PDX tumor models from individual patients before these patients develop actual systemic recurrence, which would help patients select the most appropriate course of treatment.

It has been reported that serial transplantation of tumors to recipient mice causes genetic drift [30, 31]. In general, multiple passages of PDX models or changing the site of implantation are not recommended to preserve the genetic and proteomic consistency of the original patient tumor. Serial passages might cause genomic rearrangements intrinsic to tumor adaptation [28]. This is a serious concern to use uveal melanoma cell lines established from PDX tumors for the purpose of pre-clinical drug screening to design early phase clinical trials. To minimize genetic and proteomic alterations, we orthotopically implanted liver tumors into the equivalent organ in mice. In 10 successful cases, both the original tumors and the corresponding xenograft tumors show the same tissue histologic features in H&E staining and immunohistochemistry, mutations and CNV in genomic analyses, and RPPA in proteomic analyses. Therefore, SOI with hepatic metastasis is a reliable method to create PDX mouse models that retain the characteristics of original patient tumors. Furthermore, it is preferable to restrict PDX models to a low passage number to maintain tumor characteristics of the original tumor [31]. In this regard, biobanking of PDX tumors will be critical to store patient tumors and their corresponding xenograft tumors for re-implantation when required [32]. Our preliminary studies indicate that re-implantation of cryopreserved tumors is feasible without changing the characteristics of PDX tumors. This cryopreservation approach will facilitate the expansion of our PDX platform for large-scale pre-clinical drug screening using PDX tumors.

Cytogenetic investigations have revealed that most uveal melanomas have abnormal chromosomes 1, 3, 6 and 8 [33, 34]. Monosomy 3 is observed in 50% of uveal melanoma, and 70% of patients with monosomy 3 die of metastases within four years after the initial diagnosis, whereas patients with disomy 3 (normal chromosome 3) rarely develop metastatic uveal melanoma [35]. Recently, researchers have identified mutations in BAP1 gene, located on chromosome 3, and this gene seems to play a major role in tumor progression in uveal melanoma [36]. Also, a gene expression profiling (GEP) assay demonstrated that class 2 is the most accurate poor prognostic marker for patients with uveal melanoma [37, 38]. A previous PDX study reported that tumors from patients having monosomy 3 or class 2 in GEP were relatively easy to engraft into mice [39]. However, in our study, monosomy 3 and BAP1 mutations did not clearly correlate with the success rates of engraftment and serial passage. On the other hand, high expression of Ki67 contributed to the success of serial passages. Ki67 positivity is reported to be strongly associated with class 2 in GEP and monosomy 3 [40]. The expression of Ki67 was marginally correlated with monosomy 3 in our study (*data not shown*, p = 0.0556). Ki67 has an important role in cell proliferation and tumor progression in uveal melanoma. The relative expression level of Ki67 might, therefore, be a useful parameter for predicting the establishment of a given uveal melanoma tumor in our PDX platform.

A major drawback of orthotopic hepatic PDX models is difficulty in measuring tumor growth. In this regard, we confirmed that CT scan is exceedingly helpful in evaluating tumor growth in the liver. The use of commercially available CT contrast agent allows detection and visualization of interior liver tumors in live animals. The contrast agent specifically enhances normal mouse liver parenchyma on the CT so that it is easy to recognize the unenhanced site as the tumor [41]. It also detects small tumors less than 1 mm, which arise as daughter nodules around the main tumors. This imaging approach will be highly beneficial for testing new agents for cancer treatment by accurately measuring tumor size, which is essential in human clinical trials.

Our model has some limitations that we expect to address in future work. First, xenograft tumors are surrounded by mouse tissue, even though they are orthotopically implanted. Patient-derived xenograft tumors are thus destined to be fostered by mouse vessels and tissues. Liver metastasis mouse models using human uveal melanoma cell lines contain mouse-derived vessels within the tumor [42]. Since growth factors, cytokines, and chemokines could have species-specificity, PDX mouse models might not be the best model to investigate interactions between the tumor and its microenvironment. In this regard, a chimeric mouse model with a humanized liver would provide a better human tissue microenvironment for the implanted human tumors to the liver [43]. Another major challenge to the PDX approach is the lack of tumor-immune system interactions. A humanized immune system mouse, such as a hu-BLT and hu-PBMC mouse, might be preferable for some aspects of future PDX research [44, 45].

## Conclusions

In conclusion, orthotopic PDX models can be developed from hepatic metastases of uveal melanoma patients with the liver pocket method. With CT scan imaging technology, development and growth of hepatic tumors in NSG mice can be easily monitored. This is considered to be much more close to the clinical situation of actual patients, compared to ectopic transplantation of tumor specimens to subcutaneous site devoid of liver microenvironment. Pre-clinical investigation of specific signal blockades using this orthotopic PDX model should be warranted.

## Methods

### Regulatory and ethical considerations

Patients provided written consent allowing the use of tissue samples for this research, according to an Institutional Review Board-approved protocol. The animal study was approved by the Institutional Animal Care and Use Committee of Thomas Jefferson University and adhered to the recommendations in the National Institutes of Health Guide for the Care and Use of Laboratory Animals.

### Metastatic uveal melanoma cell lines

TJU-UM001 and TJU-UM004 cell lines were established in our laboratory at Thomas Jefferson University and authenticated by the DDC Medical (Fairfield, OH, USA). They are derived from a liver metastasis and an orbital metastasis of human uveal melanoma, respectively. Both UM001 and UM004 cells harbor the Q209L mutation as determined by Sanger DNA sequencing, as previously described [46, 47]. UM001 cells were cultured in RPMI 1640 medium supplemented with 10% heat-inactivated FBS, 10% non-essential amino acids, 2 mM l-glutamine, 10 mM HEPES buffer, 50 IU/ml penicillin and 50 mg/ml streptomycin. UM004 cells were cultured in MEM medium containing 10% heat-inactivated FBS, 50 IU/ml penicillin and 50 mg/ml streptomycin.

### Patient-derived liver metastatic uveal melanoma samples

**Fig. 6:**
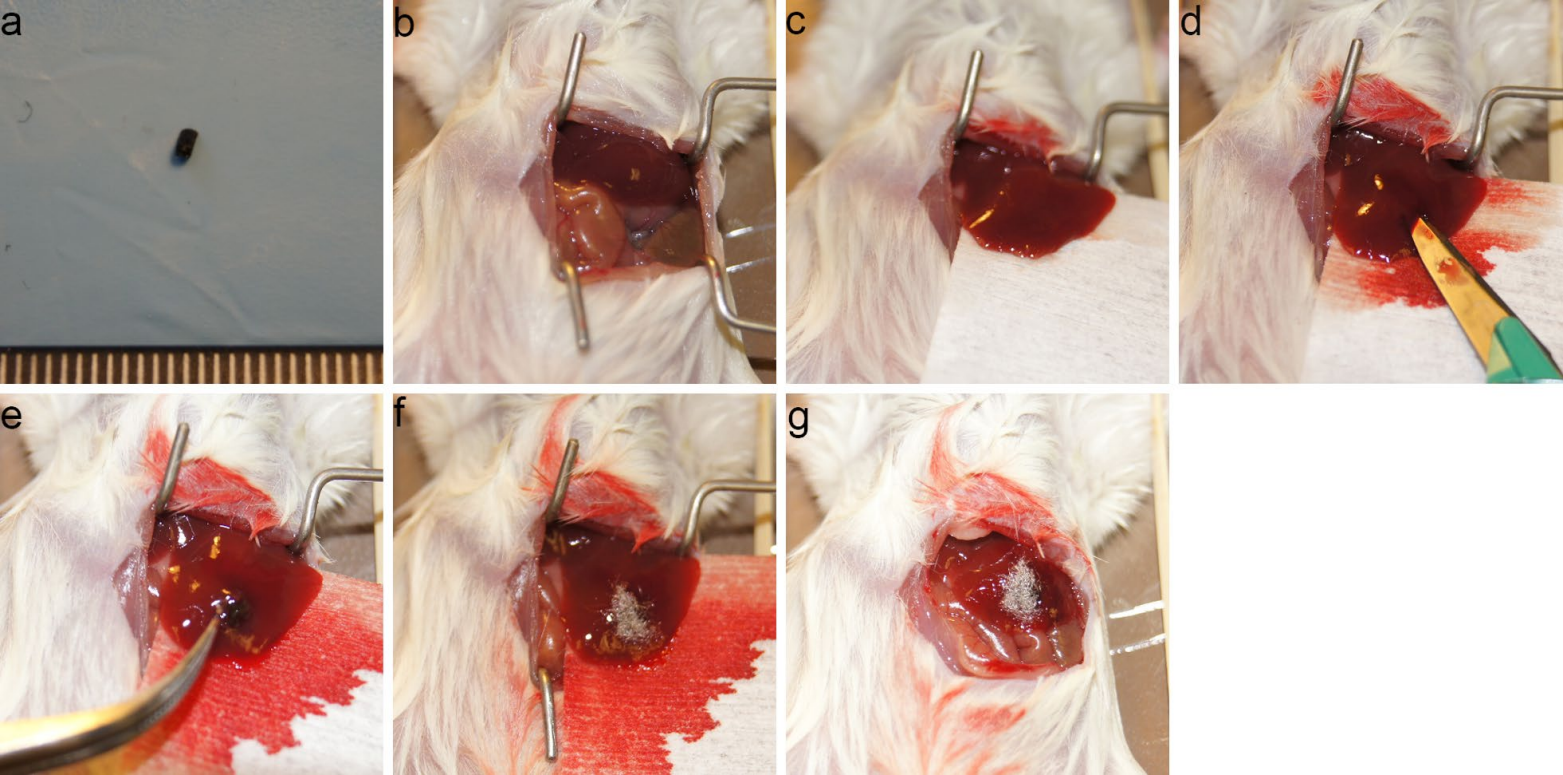
Surgical orthotopic implantation using the liver pocket method. **a** Small tumor pieces to be implanted. **b** Liver is exposed. **c** Liver is moved outside the abdominal cavity. **d** Liver pocket is produced by surgical incision. **e** Tumor piece is inserted into the liver. **f** Incision is covered and bleeding is halted using hemostatic materials. **g** Liver is returned into the abdominal cavity

Tumor specimens were obtained after surgery or biopsy from liver metastatic uveal melanoma patients. Tumor masses were washed with sterile PBS and were cut into 1 mm cubes for implantation into the mouse liver (Fig. 6a). Procurement of tumor specimens and tumor implantation were performed within 2 h.

### Animals

Eight-week-old male and female NSG mice (Jackson Laboratory, Bar Harbor, ME, USA) were used for tumor injection or implantation into the liver or a subcutaneous site. For the tumor injection or implantation, each mouse was anesthetized with 3% Isoflurane for induction and 2% for maintenance.

### Laparotomy

Mice were placed on a heating pad in the supine position. A 1 cm skin incision was made in the left subcostal area, followed by a 1 cm incision in the peritoneum to expose the liver. Using a cotton swab, the left lobe of the liver was moved outside the body and placed on a nonwoven absorbent fabric sheet for the injection or implantation. After the injection or surgical implantation, the liver was returned within the body, and the abdominal incision was closed in 2 layers with 5-0 polydioxanone absorbable thread (AD Surgical, Sunnyvale, CA, USA).

### Needle tumor injection into the liver and subcutaneous site

UM001 cells (1 × 10^6^) in 20 μl of RPMI 1640 or UM004 cells (1 × 10^6^) in 20 μl of MEM were mixed with Matrigel (BD Biosciences, Bedford, MA, USA) (mixing ratio of 2:1) and injected directly under the surface of the left lobe of the liver with a Hamilton syringe using a 27 G needle (Hamilton Company, Reno, NV, USA) [42]. For subcutaneous injection, UM001 cells or UM004 cells (1.0–10.0 × 10^6^) were injected with a 25 g needle into a subcutaneous site in the hind limb. The subcutaneously injected tumors were measured up to 12 weeks by caliper.

### Surgical orthotopic implantation using liver pocket method

The left lobe of the mouse liver was exposed by laparotomy (Fig. 6b, c). The liver was incised using a No. 11 sharp scalpel (AD Surgical, Sunnyvale, CA, USA) horizontally in parallel with the surface of the liver to form a pocket in the parenchyma without cutting any major vessels (Fig. 6d). A tumor piece was implanted into the pocket (Fig. 6e). The incision site was then sealed with absorbable hemostatic material (SURGICEL, Johnson and Johnson, New Brunswick, NJ, USA) to curtail bleeding (Fig. 6f) [48]. The liver was returned to its original position after confirming hemostasis from the liver (Fig. 6g).

### Surgical implantation at subcutaneous sites

A 3-mm incision was made in the skin of the hind limb. Scissors were inserted into the subcutaneous space to create a pocket. A tumor piece was inserted into this subcutaneous pocket, and then the incision was closed with 5-0 polydioxanone absorbable thread.

### CT imaging and contrast agent

Micro-CT scan (Inveon Micro-CT, Siemens, Germany) was performed 4 h after injection of contrast agent. The contrast agent (ExiTron nano 12000, Miltenyi Biotec, Germany) is an alkaline earth-based nanoparticulate contrast agent for mouse liver CT imaging [41]. Upon intravenous injection, the agent is taken up by cells of the reticuloendothelial system, including macrophages within the liver, termed Kupffer cells. Mice were injected with 100 µl of agent (per mouse, 25–30 g body weight) via a lateral tail vein.

### Collection of tumor masses from mice

UM001 tumors, UM004 tumors, or patient-derived xenograft tumor masses were collected from the mice at the scheduled time of sacrifice. The tumor volume was calculated using the following formula: ${\text{Volume}} = \left( {{\text{W}}^{ 2} \times {\text{L}}} \right)/ 2$, with W being the shortest diameter and L being the longest diameter. Resected tumors were washed with sterile PBS. A portion of the tumor was cut into 1 mm cubes for implantation into the liver of recipient mice. The remaining tumor was used for analyses of tumor characteristics and cryopreservation.

### Cryopreservation and thawing procedure

Tumor masses from established PDX mouse models were cut into 2 mm cubes and transferred to sterile cryotubes containing one of 3 different cryomedia: F medium (90% fetal bovine serum/10% DMSO), D medium (70% DMEM/20% fetal bovine serum/10% DMSO), and R medium (70% RPMI/20% fetal bovine serum/10% DMSO). Cryotubes were placed in a freezing chamber containing isopropanol within a −80 °C freezer, which froze the samples at a rate of −1 °C/min. Frozen tumor specimens were transferred to liquid nitrogen storage the next day. For thawing, cryotubes were incubated in a water bath (37 °C) until melted. Thawed tumors were washed with sterile PBS and then cut into 1 mm cubes for implantation into the liver of recipient mice.

### Histology and immunohistochemistry

For histopathological evaluation, H&E staining was performed on paraffin-embedded tumor tissue sections. The degree of pigmentation of tissue samples was assessed based on the Collaborative Ocular Melanoma Study (COMS) classification system [49]. For immunohistochemistry, sections were stained with primary antibodies overnight at 4 °C. On the next day; sections were incubated for 30 min in ImmPRESS AP Reagent (Vector Laboratories, Burlingame, CA, USA), followed by incubating for 2–15 min in ImmPACT NOVA-RED (Vector Laboratories). The following primary antibodies were purchased from Agilent (Santa Clara, CA, USA): Melanosome (Clone HMB45), Melan-A, S100, and Ki67. All sources of specific antibodies for these markers and their dilution rates have been previously described [42] except for Ki67 (dilution rate; 1:50). Staining data for Ki67 were quantitated by counting all positive nuclei per field of vision at ×400 magnification using ImageJ software (available at http://rsb.info.nih.gov/ij) [40].

### Reverse phase protein arrays (RPPA)

Harvested tumor tissues were lysed and processed according to the MD Anderson RPPA Core Facility protocol (available at https://www.mdanderson.org/education-and-research/resources-for-professionals/scientific-resources/core-facilities-and-services/index.html). Protein was isolated from tumor chunks, adjusted to 1 to 1.5 mg/ml, boiled for 5 min after the addition of 4× SDS sample buffer, stored at −80 °C, and submitted to the MD Anderson RPPA Core Facility. Lysates were tested using a panel of 299 validated antibodies, and analyses were performed on normalized data. Unsupervised hierarchical cluster analysis based on Pearson distance combined with average linkage was performed using MeV 4.8 (Multiple Experiment Viewer) (Dana-Farber Cancer Institute, Boston, MA, USA) [50].

### Genomic studies detecting mutations and DNA copy number variants

DNA was extracted from the tumor samples with QIAmp DNA Mini Kit (Qiagen, Valencia, CA). Genomic libraries were prepared using extracted DNA and customized for the Truseq Amplicon Cancer Panel Kit (Illumina, San Diego, CA, USA). The mutational status of GNAQ, GNA11, BAP1, SF3B1, and E1F1AX genes was assessed using the Miseq sequencer (Illumina) [51]. Specific mutations and primers for all of these genes are detailed in Additional file 7: Table S2. Genomic DNA samples were analyzed for reproducibility of genome-wide copy number, loss of heterozygosity, and somatic mutations using a single nucleotide polymorphism (SNP) microarray provided by the Affymetrix CytoScan HD Array platform (Affymetrix, Santa Clara, CA). Data analysis was performed using the Chromosome Analysis Suite software package (Affymetrix). Copy number gains and losses >1 MB are reported in this analysis. Genomic studies were performed at Cancer Genome Core Facility of Sideny Kimmel Cancer Center at Thomas Jefferson University (Director, Dr. Paolo Fortina). Unsupervised hierarchical cluster analysis based on Euclidean distance combined with complete linkage was performed using the hclust function in R version 3.3.0 (available at http://www.r-project.org).

### Statistical analysis

Data with non-normal distributions are presented with median and range. The non-parametric Mann–Whitney *U*-test was performed for comparison of Ki67 expression levels. The Pearson correlation test was performed to assess consistency of two samples in RPPA analysis and to examine correlations between Ki67 expression and tumor volume. A regression line between two samples was generated, and the correlation coefficient (r) was calculated. Fisher’s exact test was performed to identify predictive factors for the success of engraftment and serial passages. Groups were considered to be significantly different at p < 0.05. Statistical analyses were performed by GraphPad Prism (GraphPad Software, La Jolla, CA, USA).

## Additional files

**Additional file 1: Table S1.** Results of orthotopic tumor implantation to the liver using hepatic tumor specimens derived from metastatic uveal melanoma cell lines.

**Additional file 2: Figure S1.** **a–p:** Macroscopic, histopathological, and radiological features of liver-implanted tumors and subcutaneously implanted tumors using metastatic uveal melanoma cell lines. **a, i:** Laparotomy image. **b, g, j, o:** Macroscopic findings for resected tumors. **c, k:** Cut surface of the tumor. **d, l:** Sagittal imaging on CT, Black arrows indicate tumors. **e, m:** H&E staining, x10, Scale bar, 1 mm. **f, n:** Macroscopic findings for subcutaneously implanted sites. **h, p:** H&E staining, x40, Scale bar 500 µm. Abbreviation: SC = subcutaneous site.

**Additional file 3: Figure S2.** **a:** Representative biomarker expression patterns in donor and recipient tumors. Immunostaining with four antibodies as indicated. Donor sections are depicted in the columns 1 and 3; recipient sections are depicted in the columns 2 and 4. Control panels exhibit no signal with isotype control antibody, as shown by the inset images at bottom right in each immunostaining panel, x400. Scale bar 50 µm. **b and c:** RPPA correlation between donor tumors and tumors developed in the recipient mice using UM001 and UM004 cells. The scatterplot with a linear regression line shows a linear association between the donor tumors and the recipient tumors. **b:** y = 1.002 x + 0.018, r = 0.9560, r^2^ = 0.914, p < 0.001. **c:** y = 1.085 x + 0.026, r = 0.9349, r^2^ = 0.874, p < 0.001.

**Additional file 4: Figure S3.** Macroscopic and radiological features of eight PDX models. **Left panels:** Laparotomy. **Right panels:** Axial images of CT scan. Black arrows denote patient-derived xenograft tumors.

**Additional file 5: Figure S4.** Histopathological features of patient tumors and corresponding xenograft tumors in three different PDX models. H&E-stained sections and immunostained sections with HMB-45, Melan A, and S-100 antibodies, x400. Scale bar 50 µm. Control panels show no immunostaining with an isotype control antibody (inset image at bottom right in each stained panel). Abbreviations: X_0_ = Patient original tumors ; X_1_ = PDX tumors in the first-generation mice; X_2_ = PDX tumors in the second-generation mice; F = F medium (90% fetal bovine serum/10% DMSO); D = D medium (70% DMEM/20% fetal bovine serum/10% DMSO); R = R medium (70% RPMI/20% fetal bovine serum/10% DMSO).

**Additional file 6: Figure S5.** DNA copy number variation with karyogram. Individual chromosomes are shown in the karyograms, with bars on the right side of the karyograms indicating the chromosomal locations of copy number losses and gains, respectively. Chromosomes 1 to 12 are lined up on the top and chromosome 13 to 22, X and Y are on the bottom. Patient tumor (X_0_) is at the left with its adjacent karyogram, the corresponding first-generation xenograft tumor (X_1_) is in the second column, the corresponding second-generation xenograft (X_2_) is in the third column, and three different xenograft tumors generated from frozen tumor specimens using F, D and R cryopreservation medium are in the fourth to sixth columns. A copy number of 2 (normal) is indicated by blank space (no color); copy number greater than 2 (chromosomal gain or amplification) is indicated in blue; and copy number less than 2 (chromosomal loss or deletion) is indicated in red.

**Additional file 7: Table S2.** Primers used for amplification and sequencing of genomic DNA.

## Abbreviations

PDX: patient-derived xenograft
SOI: surgical orthotopic implantation
NSG: NOD.Cg-Prkdcscid Il2rgtm1Wjl/SzJ
RPPA: reverse phase protein array
H&E: hematoxylin and eosin
CNVs: copy number variations
PDOX: patient-derived orthotopic xenograft
GEP: gene expression profiling
COMS: Collaborative Ocular Melanoma Study
SNP: single nucleotide polymorphism
