# Supplementary material for: Establishment of an orthotopic patient-derived xenograft mouse model using uveal melanoma hepatic metastasis
Source: J Transl Med. 2017 Jun 23;15:145. doi: 10.1186/s12967-017-1247-z (PMC5481921; doi:10.1186/s12967-017-1247-z)
Supplement: Supplementary file 1 — Additional file 1: Table S1. Results of orthotopic tumor implantation to the liver using hepatic tumor specimens derived from metastatic uveal melanoma cell lines. [file 12967_2017_1247_MOESM1_ESM.doc]

Additional file 1: Table S1. Results of orthotopic tumor implantation to the liver using hepatic tumor specimens derived from metastatic uveal melanoma cell lines.

| Surgical Orthotopic Implantation  using Liver Pocket Method | |
| --- | --- |
| Number of operation cases | 20 |
| Surgical success rate | 100% (20/20) |
| Operation time  (Median, Range) | 22 min, 20 - 26 min |
| Bleeding during the operation (Median, Range) | 0.13 g, 0.09 - 0.20 g |
| Adverse event | 0% (0/20) |
| Operation-related death | 0% (0/20) |
