# Supplementary material for: Establishment of an orthotopic patient-derived xenograft mouse model using uveal melanoma hepatic metastasis
Source: J Transl Med. 2017 Jun 23;15:145. doi: 10.1186/s12967-017-1247-z (PMC5481921; doi:10.1186/s12967-017-1247-z)
Supplement: Supplementary file 2 — Additional file 2: Figure S1. a–p: Macroscopic, histopathological, and radiological features of liver-implanted tumors and subcutaneously implanted tumors using metastatic uveal melanoma cell lines. a, i: Laparotomy image. b, g, j, o: Macroscopic findings for resected tumors. c, k: Cut surface of the tumor. d, l: Sagittal imaging on CT, Black arrows indicate tumors. e, m: H&E staining, x10, Scale bar, 1 mm. f, n: Macroscopic findings for subcutaneously implanted sites. h, p: H&E staining, x40, Scale bar 500 µm. Abbreviation: SC = subcutaneous site. [file 12967_2017_1247_MOESM2_ESM.pdf]

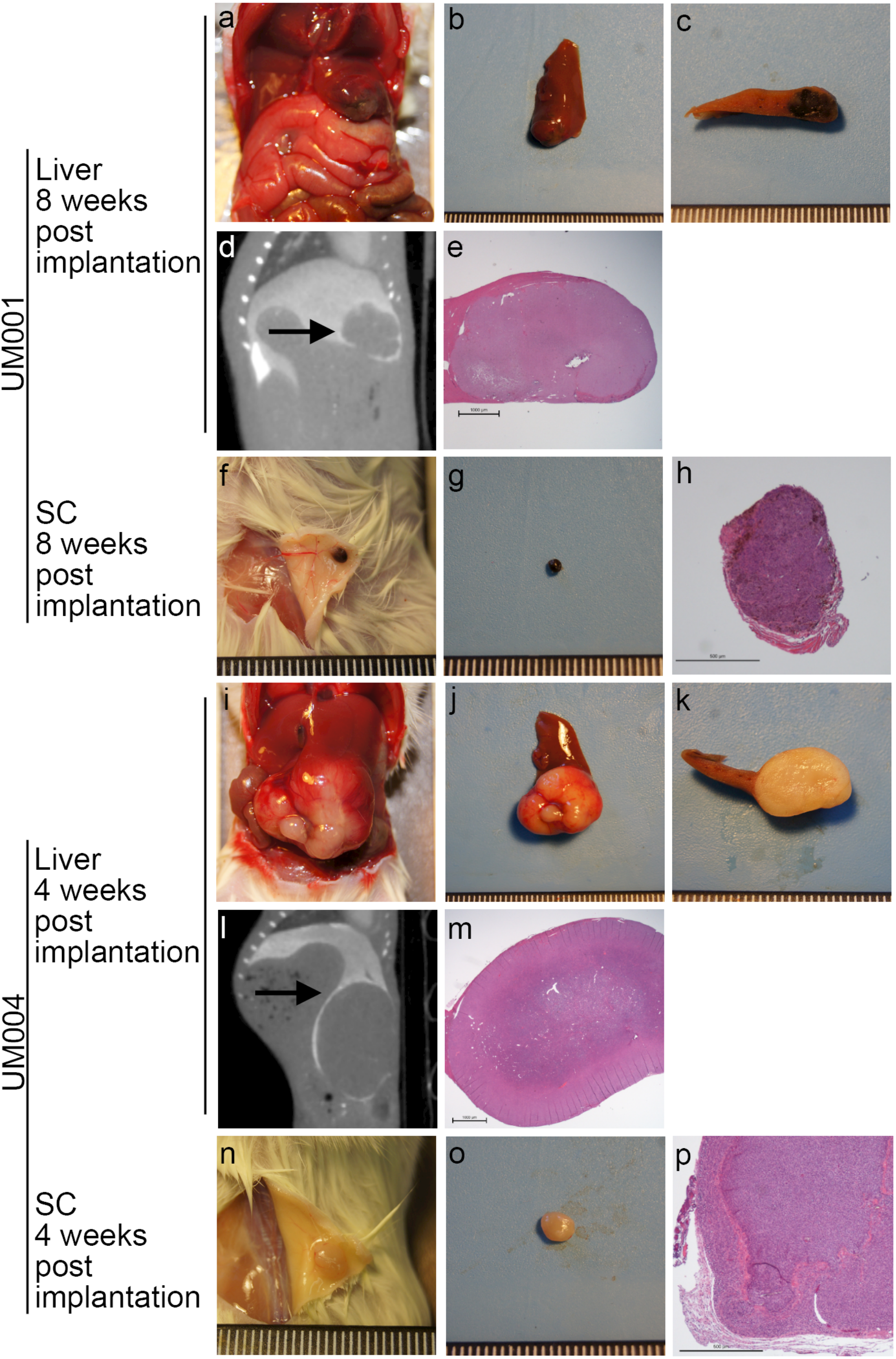
UM001
Liver
8 weeks
post
implantation
SC
8 weeks
post
implantation
UM004
Liver
4 weeks
post
implantation
SC
4 weeks
post
implantation
a
b
c
d
e
f
g
h
i
j
k
l
m
n
o
p
