# Supplementary figures and images for: Establishment of an orthotopic patient-derived xenograft mouse model using uveal melanoma hepatic metastasis
Source: J Transl Med. 2017 Jun 23;15:145. doi: 10.1186/s12967-017-1247-z (PMC5481921; doi:10.1186/s12967-017-1247-z)

**a**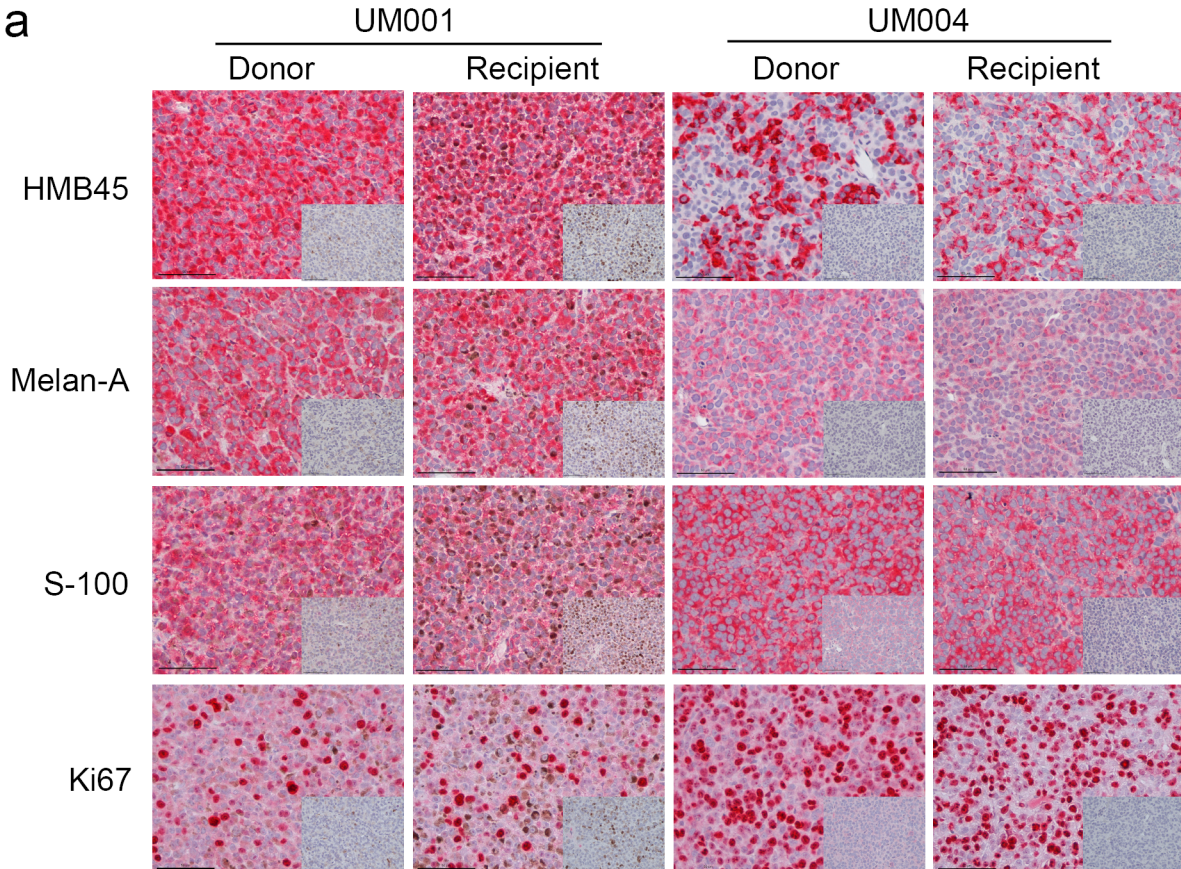**b**

UM001 donor vs recipient (Log2 scale)

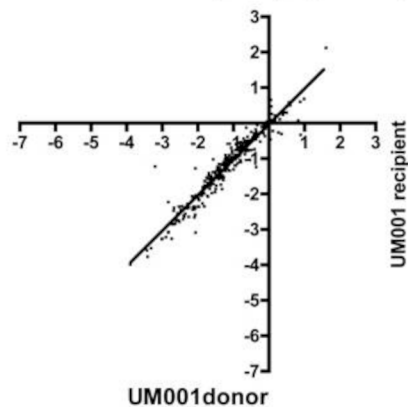**c**

UM004 donor vs recipient (Log2 scale)

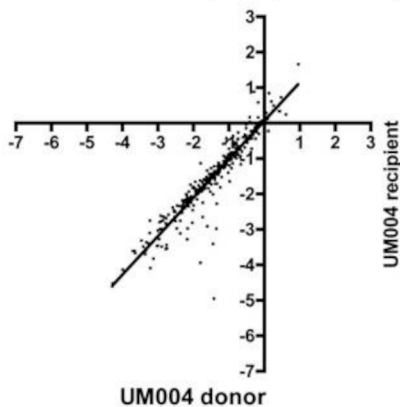

Supplement: Supplementary file 3 — Additional file 3: Figure S2. a: Representative biomarker expression patterns in donor and recipient tumors. Immunostaining with four antibodies as indicated. Donor sections are depicted in the columns 1 and 3; recipient sections are depicted in the columns 2 and 4. Control panels exhibit no signal with isotype control antibody, as shown by the inset images at bottom right in each immunostaining panel, x400. Scale bar 50 µm. b and c: RPPA correlation between donor tumors and tumors developed in the recipient mice using UM001 and UM004 cells. The scatterplot with a linear regression line shows a linear association between the donor tumors and the recipient tumors. b: y = 1.002 x + 0.018, r = 0.9560, r2 = 0.914, p < 0.001. c: y = 1.085 x + 0.026, r = 0.9349, r2 = 0.874, p < 0.001. [file 12967_2017_1247_MOESM3_ESM.pdf]

Case 4 ( $X_1$ )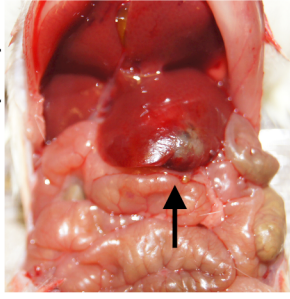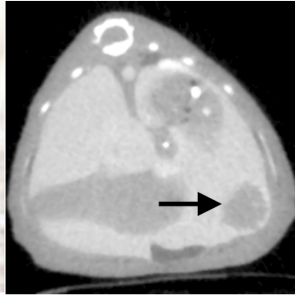Case 5 ( $X_1$ )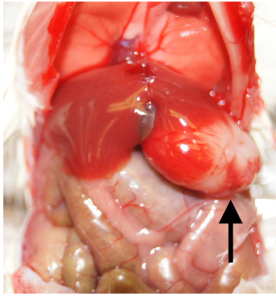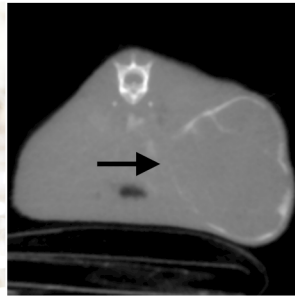Case 7 ( $X_1$ )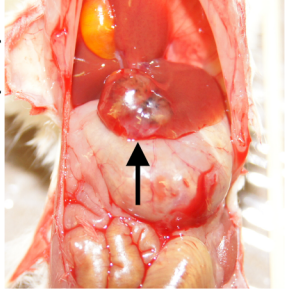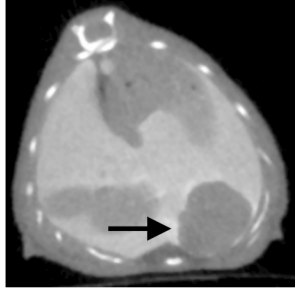Case 8 ( $X_1$ )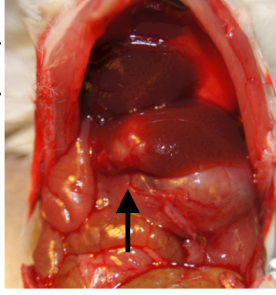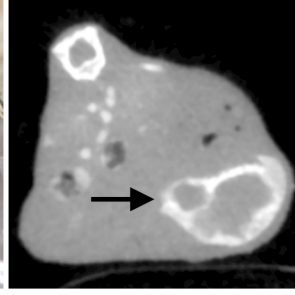Case 9 ( $X_1$ )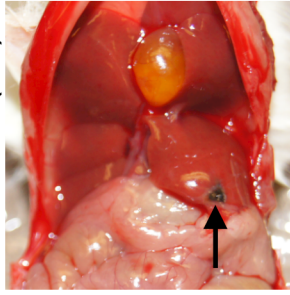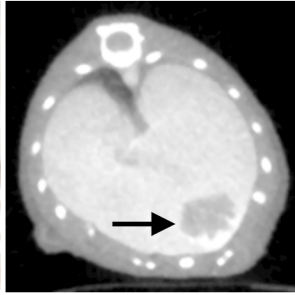Case 10 ( $X_1$ )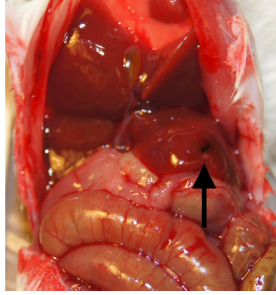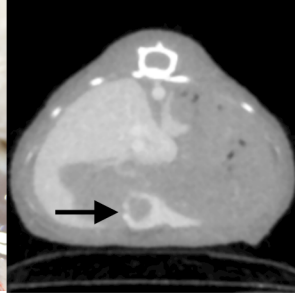Case 11 ( $X_1$ )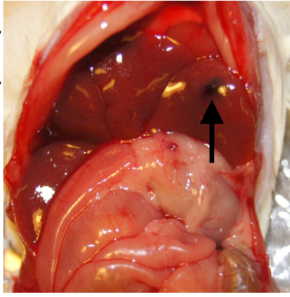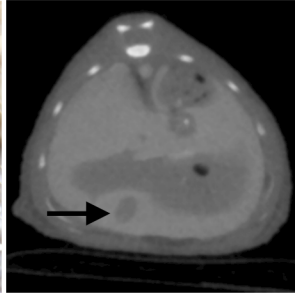Case 12 ( $X_1$ )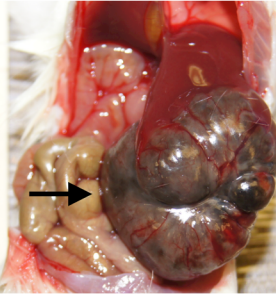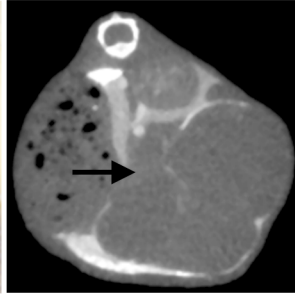

Supplement: Supplementary file 4 — Additional file 4: Figure S3. Macroscopic and radiological features of eight PDX models. Left panels: Laparotomy. Right panels: Axial images of CT scan. Black arrows denote patient-derived xenograft tumors. [file 12967_2017_1247_MOESM4_ESM.pdf]

## Case 2

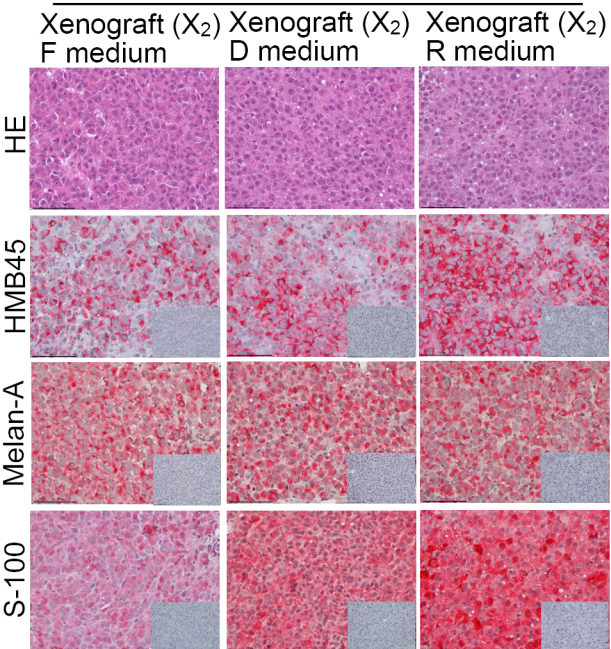

## Case 4

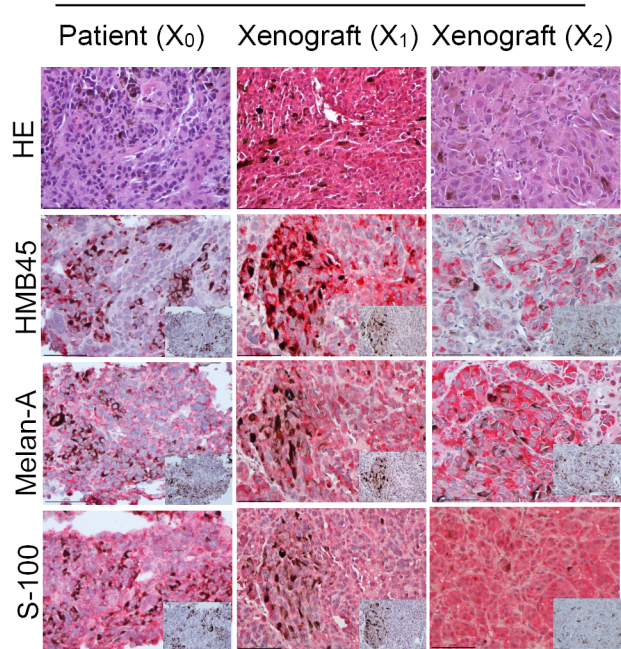

## Case 5

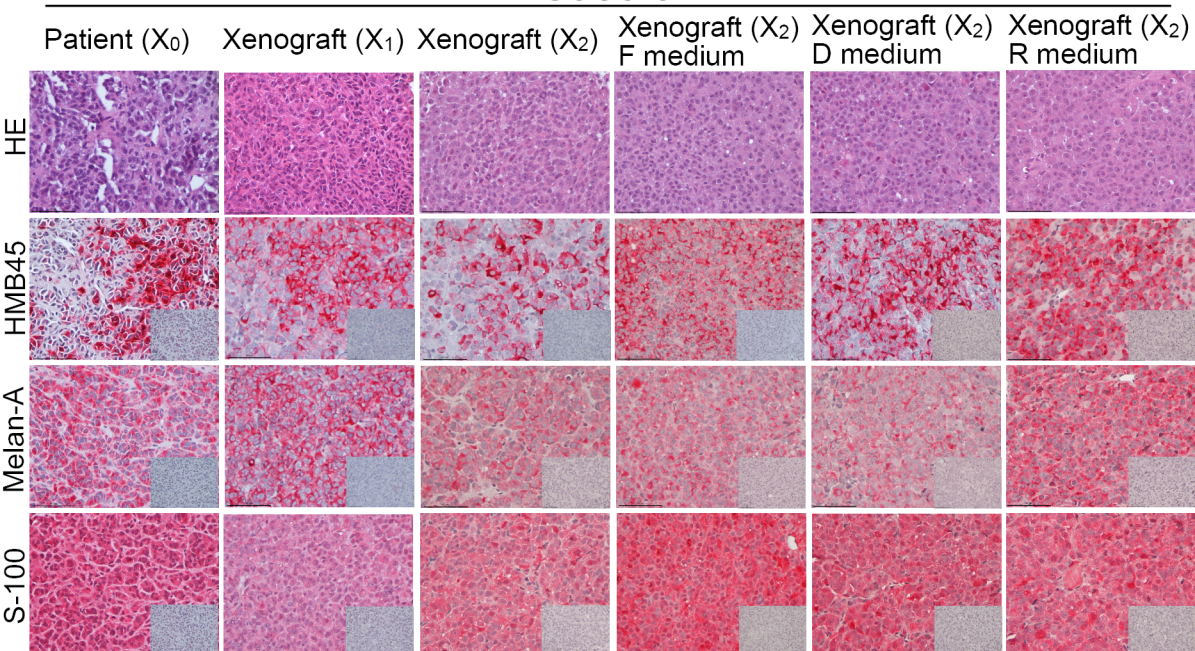

Supplement: Supplementary file 5 — Additional file 5: Figure S4. Histopathological features of patient tumors and corresponding xenograft tumors in three different PDX models. H&E-stained sections and immunostained sections with HMB-45, Melan A, and S-100 antibodies, x400. Scale bar 50 µm. Control panels show no immunostaining with an isotype control antibody (inset image at bottom right in each stained panel). Abbreviations: X0 = Patient original tumors ; X1 = PDX tumors in the first-generation mice; X2 = PDX tumors in the second-generation mice; F = F medium (90% fetal bovine serum/10% DMSO); D = D medium (70% DMEM/20% fetal bovine serum/10% DMSO); R = R medium (70% RPMI/20% fetal bovine serum/10% DMSO). [file 12967_2017_1247_MOESM5_ESM.pdf]

Case 3

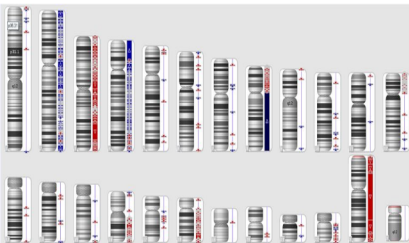

Case 4

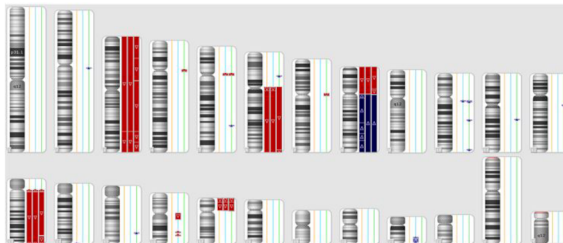

Case 5

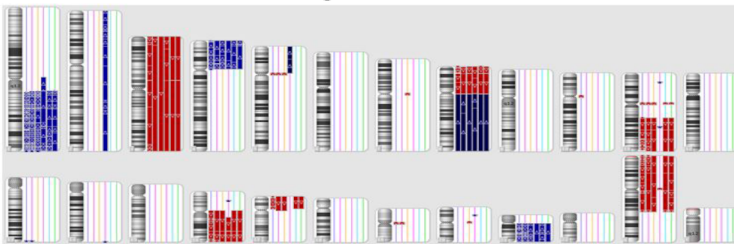

Case 6

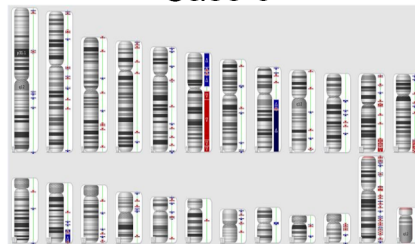

Case 7

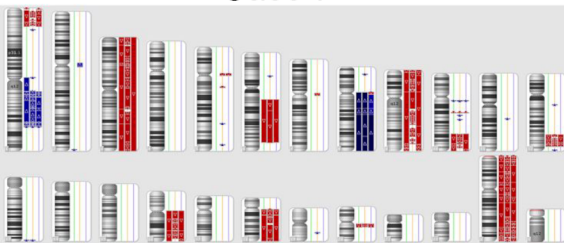

Case 8

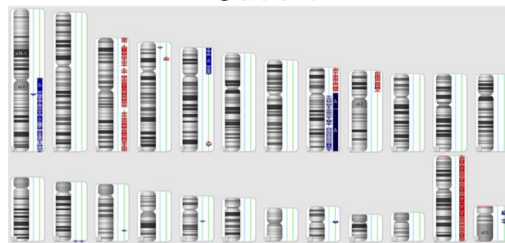

Case 9

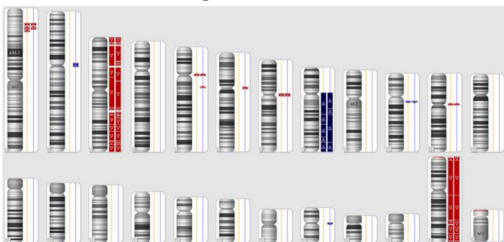

Case 10

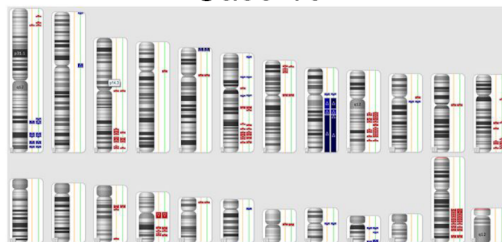

Case 11

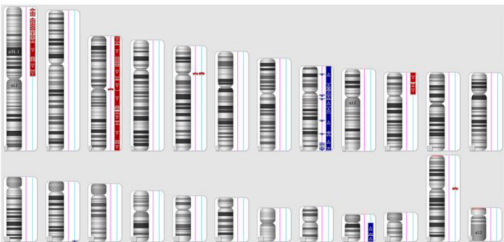

Case 12

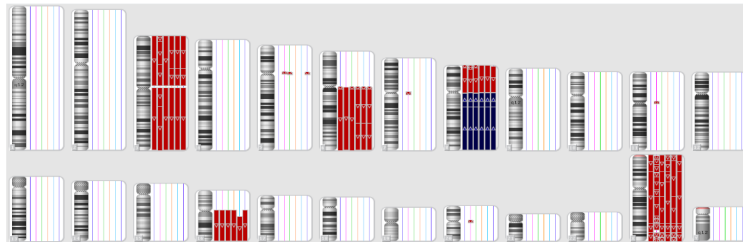

Supplement: Supplementary file 6 — Additional file 6: Figure S5. DNA copy number variation with karyogram. Individual chromosomes are shown in the karyograms, with bars on the right side of the karyograms indicating the chromosomal locations of copy number losses and gains, respectively. Chromosomes 1 to 12 are lined up on the top and chromosome 13 to 22, X and Y are on the bottom. Patient tumor (X0) is at the left with its adjacent karyogram, the corresponding first-generation xenograft tumor (X1) is in the second column, the corresponding second-generation xenograft (X2) is in the third column, and three different xenograft tumors generated from frozen tumor specimens using F, D and R cryopreservation medium are in the fourth to sixth columns. A copy number of 2 (normal) is indicated by blank space (no color); copy number greater than 2 (chromosomal gain or amplification) is indicated in blue; and copy number less than 2 (chromosomal loss or deletion) is indicated in red. [file 12967_2017_1247_MOESM6_ESM.pdf]
