# Supplementary material for: Establishment of an orthotopic patient-derived xenograft mouse model using uveal melanoma hepatic metastasis
Source: J Transl Med. 2017 Jun 23;15:145. doi: 10.1186/s12967-017-1247-z (PMC5481921; doi:10.1186/s12967-017-1247-z)
Supplement: Supplementary file 7 — Additional file 7: Table S2. Primers used for amplification and sequencing of genomic DNA. [file 12967_2017_1247_MOESM7_ESM.doc]

Additional file 7: Table S2. Primers used for amplification and sequencing of genomic DNA

| Gene | Chromosome | **Exon** | Sequence of the upstream primer | Sequence of the downstream primer |
| --- | --- | --- | --- | --- |
| SF3B1 | 2 | 1 | TTTGGCGCACGCGCAGAGTGCAGCCC | TCCCTGCTTACGTGTTTTCTTCGTTG |
| SF3B1 | 2 | 2 | TCACTTCCACCATAAATTTCCTGGTCA | TTTCCAAAAGAACTCCCAACACAATGC |
| SF3B1 | 2 | 2 | AGTGGGCCTCGATTCTACAGGTTATT | TTTTGAGAGAGCAAGAAATATGAATAACT |
| SF3B1 | 2 | 3 | CAAGCAAACTCGTAGATGATGAATAGTC | GCCCTCCTTCATAAAACTTAGAAACAC |
| SF3B1 | 2 | 3 | GGGGGTTTTCATTGCAGGATGA | GCATAGTTTATTCAGCTAAAGAAAAGTGA |
| SF3B1 | 2 | 4 | CTGGGGAAATTATCATGGTCCGCCTAT | ACAAAAATCAGAACACCATAAAGCACA |
| SF3B1 | 2 | 4 | GCAGACCGGGAAGATGAATACAAAAAG | GGCATGCTTCTGAATTTGCTTTTCTTT |
| SF3B1 | 2 | 5 | GGGTTTTCCCTCCTGCAGAAAAGAA | ATCTTACTTTTTATTGAGTTTGCTGATCA |
| SF3B1 | 2 | 5 | TTATTTATTGTACTCTTTTTCCTGTTGCT | GGTTGTGAAAGAATATAAATACAGTTGTG |
| SF3B1 | 2 | 6 | CACCAGGAGTCTGATCAGCTGTTTGAT | AGTGAGTATTTACCTTTAAAATGCTTTCA |
| SF3B1 | 2 | 6 | TCCATCAAAACGAAAACGGCGTTGG | TTGGGTTGCCATAGCAAGATACCACAG |
| SF3B1 | 2 | 7 | CACCTCGTCCAGGAGTAGCAGCT | AAAATTTTATTTCACACACCCACACAG |
| SF3B1 | 2 | 7 | ATCCTACACCTAGCCACACACCAGCGG | GAGTATGGGTGTGTGGGTGAAATGTT |
| SF3B1 | 2 | 8 | CAATTGGTGTCTTTCCAGGGGTCAGAA | CACTTCCATGCCCAGGAGTATCTTTA |
| SF3B1 | 2 | 8 | TAGTCAGATGGGTGGAAGCACTCCA | TTCCTTTGTGGTATTCTGTGTACTATT |
| SF3B1 | 2 | 8 | CAACTTTAAATGGGTCACCATTTTAGG | ATGGGCTGAGACTCCTCGAACAGATCG |
| SF3B1 | 2 | 9 | CAAGCCTGAAGCTGTTCAGGAGTCATA | TAAACGCAAACCAAGACTTAAATTTGT |
| SF3B1 | 2 | 9 | TGCTTTTCTTGGTAGGTCACATAATGA | TTTGAATGAGGTGGTATTTAGGATATACA |
| SF3B1 | 2 | 10 | GAAACCAGTCATACCACCCAAAGGT | AGCATAATGAACAATATTTGCACTTAAAAT |
| SF3B1 | 2 | 10 | AGCTCGAAAGCTGACAGCTACTCC | TTTGATAAACTATTGGTAAGTGATACTAGC |
| SF3B1 | 2 | 11 | CATAACATTTCAACTTCCCTTAACATTTT | TGTTAAATTGTCATGTTTGTTGCTTATTT |
| SF3B1 | 2 | 12 | GAACAAACCTTATGCACATATGGACGA | ATCAGTAATCTGACGCAATGCAGCCT |
| SF3B1 | 2 | 12 | AGGATACTGTACAAACTTGATGACTTAG | TGCACAAGTACACACCATACATGTACA |
| SF3B1 | 2 | 12 | CTCATGCTGTCTATGTAAAATGTGTGTA | AAGCTCGTGAATTTGGAGCTGGTCCTT |
| SF3B1 | 2 | 13 | TGTTTAATTCTGTACATGAGCATTTCATC | TCATTTCTAATTTGGCAAAGGTATTTACA |
| SF3B1 | 2 | 14 | AACTAAACTTCTAAGATGTGGCAAGATGG | GCAGAGGCTACAACAGCAAAAGCTCTA |
| SF3B1 | 2 | 14 | CAACAGATAGCTATTCTTATGGGCTGT | TTTGGGCTCAAGTGATCCTCCTGCTCA |
| SF3B1 | 2 | 14 | ACCAACTCATGACTGTCCTTTCTTTGT | CTGGGCATTCCTTCTTTATTGCCCT |
| SF3B1 | 2 | 15 | GCTTCAGCCAAGGCAGCAATGGCCAAA | AGCCAAACTGCAGAATATGTTCACATT |
| SF3B1 | 2 | 15 | AGCAGAAAGTTCGGACCATCAGTG | TTGATTTATCTTCATTAAAGTTAAGGCGA |
| SF3B1 | 2 | 16 | GAAGGCTATTGGGTATCTTATTCCTCTT | GTTAATGTAAACTGGATATGTTTCATGGT |
| SF3B1 | 2 | 17 | TGTAGTTTGCTTCTACACCATCTGTCC | CAAACAGTATTCGTGTAACATACAGTT |
| SF3B1 | 2 | 17 | ACCTTTTAGGTGGTAAAACAGTGTTGT | TGTAAAAGAATATTCTCAAAAGTCCTTTCA |
| SF3B1 | 2 | 18 | TAATTTTCTCAATTGTCTCCATCACCA | AAACCTTTTAGACTGCTTTTCCAAGGA |
| SF3B1 | 2 | 18 | GAAAGATGAAGCCGAACAGTACAGAAAA | ATTATGGCACATTGCTACTAACTTTAATAT |
| SF3B1 | 2 | 19 | CTGTACCACAGATCTGAGGCAAGTATG | TCAACCTTTTCTAACCACCCAAACATC |
| SF3B1 | 2 | 19 | GTTAATGCTCTTGGCAAACGAGTCAAA | TTGCATCAAATTCTTACTTCTTCCCCA |
| SF3B1 | 2 | 20 | GGGTGTTGTATTGTATGAGTATTTGGGT | AAATCTTATTTAGGAGTTGGGAGGTTT |
| SF3B1 | 2 | 21 | GAGTCTAGGCAGCAGATCTTTAATTGG | TAAACTTACAGCTGCCTAGGGAAAGAG |
| SF3B1 | 2 | 21 | CTATTAAATAGGTATGCATAAGATGACTCC | ACCCACACATTTTTATAAGCAGTATCA |
| SF3B1 | 2 | 22 | ATAGCCTTTTTGTGGGCTTTTAAGAGC | ATTTGCAAATTCAGTTCTAAAAACATGAT |
| SF3B1 | 2 | 22 | GGATGAGGATTTGCTTTGAGCTTTTAG | GCATTGTGGTATTGGAAAATACTTCCA |
| SF3B1 | 2 | 23 | AGTCACTTACCTATCCATTAAAGCATC | GGGAGTACTGTAAAGGGTGAACATGT |
| SF3B1 | 2 | 23 | AGCCAATACATCATGAGGGCTGAAA | CTTTCAAGTCACTGGCATTGTTGTTTC |
| SF3B1 | 2 | 23 | CATTTATGCCGTAACACCGTTACTTGA | AAATTGCTAGCCTGGAGTCTTAGGAC |
| SF3B1 | 2 | 23 | TGTAACAGCTTGTTGACCCATTTGTTT | GCCTTAATGAATGAATACAGAGTTCCTGA |
| SF3B1 | 2 | 24 | CAACAAGTGATTCAGCGAATCTTCACA | GGGGAAAAATCCTTAAAGATACAGTCT |
| SF3B1 | 2 | 24 | CATGTCACTTGGGGTTTATGGATTTGG | TGCAATATTGTTTACAGGTAAGTTAAAGA |
| SF3B1 | 2 | 25 | GGAACCAATGTAGATGGAGTTGTAAAT | TGATTCAAAATTATTTCCCTTGGGGAG |
| SF3B1 | 2 | 25 | CCCGGAAAGTCAGAGATGTATATTGGAA | TATTTTGTGTTTAATGCACAGCTACTT |
| SF3B1 | 2 | 10, 11 | GTTGATTCATCAACATCAACCTATAGTAA | TCATCAGGTTTTAAAAATGGAAGATTTCC |
| SF3B1 | 2 | 13, 14 | GTAGCCAGACCAGCAGCCTAAAATGTA | CTCTCGGCCTTCCACTCTAGCATAGTAAT |
| SF3B1 | 2 | 15, 16 | TATAGTAGTTGGCATATTCTGCATCCA | AGGTAATTGGTGGATTTACCTTTCCTC |
| SF3B1 | 2 | 19, 20 | TGCTGCCCAATACTTCAGGGTACTCTT | AGTTTACCTCTTGACAAGTCTTCATGAC |
| BAP1 | 3 | 1 | TCCCGGTGGGGGCAGCGGTGGGGA | ATGGGCCCGGGACGCGCCTGCCTGACC |
| BAP1 | 3 | 2 | CTGATGAGTGAGGGCGCAGGGGT | CGGCCTCCCCAGCCCCTGGCCCTC |
| BAP1 | 3 | 3 | TGCCGGACCGGGGCTGTGGCGGCC | TACACCAGGGCTGCCCCTTACACCCAG |
| BAP1 | 3 | 4 | CTCTTCGATCCATTTGAACAGGAAGAT | CCCCTCTCCCCTGGCTTCTTCCCAACA |
| BAP1 | 3 | 4 | TACTTCCCCCAGCCCTGTATATGGATT | TCTGTGCTTTGTTTGGAGGGTGG |
| BAP1 | 3 | 5 | GGCATGAGTTGCACAAGAGTTGGGTAT | TCAGCTCCTTTCATCTTTGCCTAATGT |
| BAP1 | 3 | 5 | GGGTGACTATTCTTGGTTTCACAGCT | CTTCATCCTGGCTCACAGCCAA |
| BAP1 | 3 | 6 | TGTGTTCCTTCCGATTCCTGGAATG | ATAATAGCCATGCCAGGTGTGTGGGAG |
| BAP1 | 3 | 7 | CCACGCCACCTCCCTGAGAAGC | TGGAGGCCTGTTGGGTGTCTCTG |
| BAP1 | 3 | 8 | TTCCTTCCCACTCCTGGCCTGCCCAAA | CCTGCCCTGTTCTACTCTCTGGAG |
| BAP1 | 3 | 9 | CATCAGGTTGAAGCGGATGTCGTGGTA | AACAGGCAGGCAGCGACTAGCCATACAT |
| BAP1 | 3 | 9 | TGCGGCTGTGACTGCAGGGAGCC | TTCCTGGCCTCTGCCCAGCCCAACCCTC |
| BAP1 | 3 | 10 | CTCAGGCAGCTGTGACTCTTGAGACTT | AGGAATAATGGCCTTGGCTCTACCCAT |
| BAP1 | 3 | 10 | TAACACAGCCAGAGCTGATTCAGACCCA | TTTGGGACCTCTTGTGGACCTCAGAGC |
| BAP1 | 3 | 11 | CCCCATTGAGGCTGCTGCCTGGA | ATCTCCAAGAAAAGCTCACAGTCTCC |
| BAP1 | 3 | 11 | CCAACAAACCCAAGCTAGTGGTGAA | CACCCTTGCAGGATTCTCTACTTGATTC |
| BAP1 | 3 | 12 | GCTGCGGCCCACACCTGCCGCCAGGTCTT | CCTCCAGCTGTTCCATGGCCTGTCCTT |
| BAP1 | 3 | 12 | CCTGGTCTTTGTCCCAGGAGGAA | TTTCTAAGGCTACCAGGTTCTAGGTGC |
| BAP1 | 3 | 12 | GCGTATGCAGTCAACACGCAGCA | AGGCCGTGTCTGTACTCTCATTGCT |
| BAP1 | 3 | 12 | GAGGAATTGAGAGGTCCTTCTGGGA | CAAGGGAACGGGCCAGGTGACCATA |
| BAP1 | 3 | 13 | AAGGTGCTTTTTGGAGAGGATGACAGC | TCACTGGCCACTTGGTGCACCCAGGAG |
| BAP1 | 3 | 13 | AACGTCTTGGCTGAGAAGCTCAAAGA | TGAGATCGGCAGTGCTTTCAACTCGCC |
| BAP1 | 3 | 13 | TCAGAACTTGATGCCAGGCTTAGCAT | TTCACATCTTCTCGGGCCCCACAGGTA |
| BAP1 | 3 | 14 | CCCTCCATCAGACCAATCCAAGG | TGTGGTTTTCTCCTTTAATCCTGGCA |
| BAP1 | 3 | 15 | TCATCCTTGCCTCTAGCTGCCTATTG | GGTAGAGAAGAGGAAGAAGTTCAAGGT |
| BAP1 | 3 | 16 | ATTGGCTCCAGTGCTCTCAGTCTTCT | TGTCCTTCAGCTCCCCTCCCCTG |
| BAP1 | 3 | 17 | ATGTTCTGCTCCACTAGGTTGGC | GGCCCTCAGCAGGGCATTCCAGTTAA |
| BAP1 | 3 | 17 | TCTCTACCTCTTCGCAGGCATGCT | CCTGACTCTGCAGCCCACTCTT |
| BAP1 | 3 | 13, 14 | ACTGGGCTGCTGGACCCCTGGCT | CCAGTCCAAGGCCCACCTGTCA |
| BAP1 | 3 | 15, 16 | TTCTCTGGTCATCAATCTGTAGGAGAG | CCTCCTTGAGGCACGCCTCATAGTTT |
| BAP1 | 3 | 6, 7 | ATGGTCCGCACTGCACTAAGGCCATT | GGCCTTGGCCAACTCCGGGGCATT |
| GNAQ | 9 | 1 | TCCTTGGCCTCCTCGCTCAGGCA | CCCGGGCGCGCGTCCGGGACGAGCTC |
| GNAQ | 9 | 1 | AATGACTCTGGAGTCCATCATGGCGTG | GGGCCTCTGCGCCCCCAGCCCA |
| GNAQ | 9 | 2 | CTTGGTGAAGCCCCTTTTATCTTCATC | ACCAAATACCATGCCTTGGATTTAACA |
| GNAQ | 9 | 2 | GAGAATCATCCATGGGTCAGGATACTC | CTTCATTGGAGCAGTCAGCTGAATGT |
| GNAQ | 9 | 3 | GGATTCTCAAAAGCAGACACCTTCTCC | AGGTAACACCTTCCTTCATATACTGGTC |
| GNAQ | 9 | 3 | CATGCACAATTAGTTCGAGAAGTTGATG | CAGGTGGCCCTATGGATTTTTCTCT |
| GNAQ | 9 | 4 | CGTAGGCAGGTAGGCAGGGTCAGCTA | AAAATATCAGAAAGTGGTAGAGGAGTCTG |
| GNAQ | 9 | 4 | CCTCTAGCTATCTTAATGACTTGGACCG | GTCCATGAAACCTATTTCCCAGCTTTT |
| GNAQ | 9 | 5 | AGTGTATCCATTTTCTTCTCTCTGACC | ATTCTCTTGAATGACGATGATCATCCAAG |
| GNAQ | 9 | 5 | AGAATGGTCGATGTAGGGGGCCAA | TTAGACAGGCAATGGATTATCATTTTATT |
| GNAQ | 9 | 6 | CTGGAACCAGGGGTATGTGATAATTGT | AACCCATCTTTTGTATACGACCAGTTT |
| GNAQ | 9 | 6 | ATGGAGGAAAGCAAGGCTCTCTTTAG | TCATACCACAGCTCAGTAGCTGTGAA |
| GNAQ | 9 | 7 | CGTGAAGTGGGAGTAGATAATTTTGTCAC | GGTTCAATAAATACTGTGTCATTGCTC |
| GNAQ | 9 | 7 | AAGATGTTCGTGGACCTGAACCCAGAC | TAGACACCCGCCCTGCCCTTCCCTGGT |
| GNA11 | 19 | 1 | TGACTCTGGAGTCCATGATGGCGTGTT | CGGCTGCGGGCCCTGCCCTGCCTGTG |
| GNA11 | 19 | 1 | TTGGACTCCTTCACCTCATCGCTCAGG | CTCCGGCCTCGGCCACCGGCCCTCGG |
| GNA11 | 19 | 2 | CGGCTTCACCAAGCTCGTCTACCAGAA | GTGGTGAGCATGGTGGCCGCGCT |
| GNA11 | 19 | 2 | ATCATGGCCTGCATGGCGGTGAAGAT | GCACGTGAGGGCGGGCGGCAGC |
| GNA11 | 19 | 3 | GGAGGACCCGGGCATCCAGGAATGCTA | CCTGCCTGCTCGCCGGGGGCAGGGAT |
| GNA11 | 19 | 3 | AGAGCTGGTACTCGCGCCTGCGGTC | GCAGGGGAGACGTCGAGAGCTC |
| GNA11 | 19 | 4 | CTGCCCACCCAGCAGGACGTGCT | GCTCATGCCTGCGCACCCAGTGCTTT |
| GNA11 | 19 | 4 | ATGATGCCGGTGGTGGGCACGCGGACCCG | ACAGGGACACAGCACCCAAACCAGCG |
| GNA11 | 19 | 5 | CCCTCAGCGAATACGACCAAGTC | CAGTGGGGAGGGCCCCTCTGATT |
| GNA11 | 19 | 5 | ACCTCGTTGTCCGACTCCACCA | CCAGGACTCAGCCACCTGGCGC |
| GNA11 | 19 | 6 | CTCCTCCGTCATCCTCTTCCTCAACAA | GGCTGATATGGGAGAGGGGCTCATACA |
| GNA11 | 19 | 6 | ATCTTGTCCTCCAGCAGGTCCTT | ACACAGCCCAAGGGGCAGCTGGGGG |
| GNA11 | 19 | 7 | ATCTACTCACACTTCACGTGTGCCA | CAGGACCTTCCTTCCACGGAGC |
| GNA11 | 19 | 7 | AACACGAAGCGGATGTTCTCCGTGTCG | AGAGGGTGAGGCTGTGCCCCAGCCCG |
| EIF1AX | X | 1 | TTGCCTGGCCTCCAGCACCTACTTG | GGACCCCCGGACCACGGCTCTG |
| EIF1AX | X | 2 | ACAGATAATTAATGTCATTTACCTCCTTTT | ACGCTTTTTAAAATAACATCCTTTCTTTTT |
| EIF1AX | X | 3 | GAACAATATTCCATACCCTTTTCCTTTC | ACTTTAATATAAAATTGGTTAAGGGGGAAA |
| EIF1AX | X | 4 | AACTAATTGATTGTTACTCTGGAATTTCT | TGTAAATTTTTCCTATTTTACTGGCTTGAA |
| EIF1AX | X | 5 | TTACCATGCTCTGGAAGCTCGCCGTAT | TGAATAAACAATCAAATCTCACCGTTT |
| EIF1AX | X | 5 | ATGCAGACGAAGCTAGAAGTCTGAAGG | TTTAGTTTTAAGTATGCCTGCCTATGC |
| EIF1AX | X | 6 | ACTTTGAGTAAATTACAGTGCTGACTT | ACCTTGTGTTTTCCAATTTTTGTTATTATC |
| EIF1AX | X | 7 | GCAGGCCAGAATTATAGGAATTTCTTCT | CAACATTTTACATTCCATCTTTTCTGAAG |
